# Engineering of pH-dependent antigen binding properties for toxin-targeting IgG1 antibodies using light-chain shuffling

**DOI:** 10.1016/j.str.2024.07.014

**Published:** 2024-09-05

**Authors:** Tulika Tulika, Fulgencio Ruso-Julve, Shirin Ahmadi, Anne Ljungars, Esperanza Rivera-de-Torre, Jack Wade, Monica L. Fernández-Quintero, Timothy P. Jenkins, Selma B. Belfakir, Georgina M.S. Ross, Lars Boyens-Thiele, Alexander K. Buell, Siri A. Sakya, Christoffer V. Sørensen, Markus-Frederik Bohn, Line Ledsgaard, Bjørn G. Voldborg, Chiara Francavilla, Tilman Schlothauer, Bruno Lomonte, Jan Terje Andersen, Andreas H. Laustsen

**Affiliations:** 1 Department of Biotechnology and Biomedicine, Technical University of Denmark, Lyngby, Denmark; 2 Department of Pharmacology, University of Oslo, Oslo, Norway; 3 Department of Immunology, Oslo University Hospital Rikshospitalet, Oslo, Norway; 4 Precision Immunotherapy Alliance (PRIMA), University of Oslo, Oslo, Norway; 5 VenomAid Diagnostics ApS, Lyngby, Denmark; 6 Roche Pharma Research and Early Development (pRED), Roche Innovation Center Munich, Penzberg, Germany; 7 Instituto Clodomiro Picado, Facultad de Microbiologia, Universidad de Costa Rica, San Jose, Costa Rica

**Keywords:** Antibody recycling, pH-dependent antigen binding properties, acid-switched antibodies, light-chain shuffling, phage display technology, FcRn, human endothelial cell-based recycling assay, HERA, α-cobratoxin, myotoxin II, snake venom

## Abstract

Immunoglobulin G (IgG) antibodies that bind their cognate antigen in a pH-dependent manner (acid-switched antibodies) can release their bound antigen for degradation in the acidic environment of endosomes, while the IgGs are rescued by the neonatal Fc receptor (FcRn). Thus, such IgGs can neutralize multiple antigens over time and therefore be used at lower doses than their non-pH-responsive counterparts. Here, we show that light-chain shuffling combined with phage display technology can be used to discover IgG1 antibodies with increased pH-dependent antigen binding properties, using the snake venom toxins, myotoxin II and α-cobratoxin, as examples. We reveal differences in how the selected IgG1s engage their antigens and human FcRn and show how these differences translate into distinct cellular handling properties related to their pH-dependent antigen binding phenotypes and Fc-engineering for improved FcRn binding. Our study showcases the complexity of engineering pH-dependent antigen binding IgG1s and demonstrates the effects on cellular antibody-antigen recycling.

## Highlights

• IgGs against the snake venom toxins α-cobratoxin and myotoxin II were discovered
• Light-chain shuffling was used to generate IgGs with pH-dependent binding properties
• Binding between IgG and snake venom toxins affects FcRn-mediated cellular handling
• Cellular handing of IgG-bound toxins is complex and affected by multiple factors

## Graphical abstract

**Figure:**
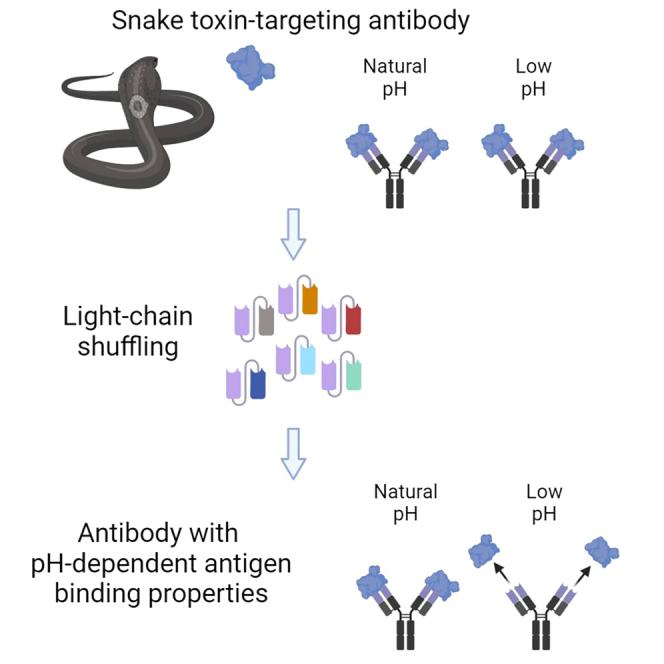

## Teaser

Tulika et al. utilized light-chain shuffling and phage display technology to generate IgG1 antibodies with pH-dependent antigen binding properties against snake venom toxins. Combined with Fc-engineering, this strategy may be useful for the development of acid-switched antibodies.

## Introduction

Monoclonal antibodies (mAbs) are used as therapeutic agents to treat a variety of diseases because of their ability to bind antigens with high specificity and affinity.^1^^,^^2^^,^^3^ The most used antibody format is built on human immunoglobulin G1 (IgG1),^2^^,^^4^ which has a plasma half-life of three weeks on average in humans due to its recycling, mediated by the pH-dependent binding interaction between its fragment crystallizable (Fc) region and the neonatal Fc receptor (FcRn).^5^^,^^6^^,^^7^ Briefly, IgGs in the bloodstream are taken up by cells via fluid-phase pinocytosis, followed by entry into endosomes where the mildly acidic pH facilitates engagement of FcRn. The FcRn-IgG complexes are then recycled back to the cell surface where exposure to the near-neutral pH of the extracellular environment triggers the release of IgGs from the receptor. As such, FcRn rescues IgGs from lysosomal degradation in a strictly pH-dependent manner, which explains the long half-life of IgGs.^8^^,^^9^^,^^10^^,^^11^ Insight into this mechanism has provided a basis for the development of Fc-engineering strategies that allow for more favorable engagement of human FcRn (hFcRn), which can be exploited to extend the plasma half-life of IgGs in hFcRn transgenic mice, non-human primates, and humans.^12^^,^^13^^,^^14^^,^^15^^,^^16^ One such Fc-engineering technology is based on three amino acid substitutions (M252Y/S254T/T256E; YTE), which increases the binding affinity of the human IgG1 Fc region to the hFcRn at acidic pH without compromising the pH dependency of the interaction, resulting in up to 4-fold extended half-life in non-human primates.^12^^,^^15^^,^^17^ In line with this, it has been demonstrated that YTE-containing IgG1s can have half-lives ranging from 80 to 112 days in humans, whereas the wild-type (WT) counterpart only has a half-life of about 20 days.^18^ However, biophysical properties of the variable domains of the antigen binding fragment (Fab) arms, such as surface charge patches and their influence on the isoelectric point (pI) of the IgG, may affect plasma half-life in both an FcRn-dependent and independent manner.^19^^,^^20^^,^^21^ Thus, to tailor IgGs for optimal pharmacokinetics, there is a need to gain an in-depth understanding of how such factors affect cellular uptake and FcRn-mediated transport, both in the absence and presence of the cognate antigen.

IgGs can bind both soluble antigens and the extracellular domain of membrane-bound antigens. These complexes may stay bound during cellular uptake and throughout the endosomal pH gradient, resulting in lysosomal degradation of both the IgGs and the antigens. Alternatively, the complexes may be recycled by FcRn to the cell surface membrane followed by exocytosis and release of the IgGs in complex with the soluble antigens into the extracellular space.^19^^,^^22^ Hence, the antigens continue to occupy the binding sites of the recycled IgGs. This means that high doses of IgGs are needed when the targeted antigens are present in large amounts.^23^ To overcome this challenge, the antigen binding properties of an IgG can be engineered in such a way that affinity is kept high at near-neutral pH but becomes weaker when pH is lowered, such as in the acidic environment of the endosomes.^13^^,^^23^^,^^24^^,^^25^^,^^26^^,^^27^^,^^28^^,^^29^^,^^30^^,^^31^ This enables the release of the antigens in the acidic endosomes followed by lysosomal degradation, whereas the IgG will be recycled in an FcRn-dependent manner and released into the extracellular space, ready to bind new antigens.^23^^,^^24^ Thus, the same IgG molecule will have the capacity to bind and direct antigens for lysosomal degradation multiple times. This strategy has been explored in the design of IgGs targeting endogenous antigens that drive chronic diseases,^13^^,^^23^^,^^24^^,^^25^^,^^26^^,^^27^^,^^28^^,^^29^^,^^30^^,^^31^^,^^32^^,^^33^^,^^34^^,^^35^ which makes it possible to achieve an equivalent therapeutic effect using lower antibody doses and/or less frequent dosing compared to when non-pH-dependent IgG counterparts are being used.^13^^,^^23^^,^^30^ Such engineered IgGs with pH-dependent antigen binding properties, also known as acid-switched antibodies, are attractive when frequent dosing and/or high doses would otherwise be required to achieve a therapeutic effect. Moreover, the use of such antibodies could also be beneficial to lower the costs of treatment, which is desirable for many indications, including autoimmune diseases, infections,^36^ and snakebite envenoming.^37^^,^^38^

The most common approach employed to engineer acid-switched antibodies involves the incorporation of histidine residues into their variable regions.^13^^,^^23^^,^^25^^,^^27^^,^^28^ The rationale behind this is that histidine has a pK_a_ of ∼6.0, which enables protonation at pH 6.0 and below, thereby potentially weakening the antibody-antigen interaction in acidified endosomes due to electrostatic changes in the paratope of the antibody.^39^^,^^40^ However, such protein engineering approaches must be tailored to not negatively affect antigen binding at neutral pH^28^ or developability, which could increase the risk of aggregation and/or immunogenicity.^41^^,^^42^^,^^43^ To circumvent these challenges, an alternative strategy that allows for the discovery of acid-switched IgGs with entirely native variable heavy (V_H_) and variable light (V_L_) domains has recently been reported.^38^ This strategy involves the use of phage display technology combined with a large naive human antibody library, which has been demonstrated to be useful for the discovery of IgGs with pH-dependent antigen binding properties, and for the identification of antibody candidates that are entirely devoid of histidine residues in their variable regions yet display strong pH responsiveness.^38^

In this study, we present a new strategy for the discovery of acid-switched IgG1 mAbs with entirely native V_H_ and V_L_ domains by combining light-chain shuffling and phage display technology. We demonstrate the utility of this approach by increasing the pH-dependent antigen binding properties of existing IgG1 mAbs targeting two snake venom toxins,^38^^,^^44^ namely α-cobratoxin (α-cbtx) from *Naja kaouthia* (monocled cobra from Southeast Asia) and myotoxin II (M-II) from *Bothrops asper* (Fer-de-Lance from Central America). These toxins were chosen as antigen since they are among the medically most important snake toxins for human health.^45^^,^^46^^,^^47^ Specifically, α-cbtx blocks the function of nicotinic acetylcholine receptors in synaptic clefts and disrupts neuromuscular transmission, which causes paralysis in victims and prey,^48^ while M-II is a phospholipase A_2_-like protein that damages cell membranes, which leads to severe tissue damage that often requires amputation.^49^^,^^50^ Using a combination of assays, we find that the developed IgG1s bind hFcRn in a pH-dependent manner and that they are therefore rescued from intracellular degradation via FcRn-mediated recycling. We further show that this latter feature could be enhanced by Fc-engineering. Importantly, in the presence of soluble toxin antigens, more of the acid-switched mAbs were recycled as free IgG1s, while those with no pH-dependent antigen binding properties were recycled in complex with their cognate antigen. However, differences in the cellular uptake and recycling of the different IgG1 clones were observed in the presence of the two different antigens. While cellular uptake, recycling, and accumulation were either reduced or not affected for the IgG1s in complex with α-cbtx, these cellular parameters were enhanced for IgG1s bound to M-II. This demonstrates that acid-switched mAbs with distinct binding kinetics are handled differently in a cellular context depending on the targeted antigen.

## Results

### Discovery of antibodies with pH-dependent antigen binding properties by light-chain shuffling

Previously, four human IgG1 mAbs binding M-II were discovered using phage display selections, in which binding of single-chain variable fragment (scFv)-displaying M13 phages to the native antigen at pH 7.4 was followed by elution of bound phages at pH 6.0.^44^ To screen if these IgG1s possessed antigen binding properties that were sensitive to pH alteration, biolayer interferometry (BLI) measurements were performed by adding the IgG1s at pH 7.4 to biotinylated M-II captured on streptavidin-coated biosensors before dissociation was performed at either pH 7.4 or pH 5.5 (Table 1). The results revealed that two of the IgG1s, TPL0039_05_A03 and TPL0039_05_E02 (hereafter referred to as A03 and E02), displayed similar relative off-rates at both pHs, while TPL0039_05_B04 and TPL0039_05_B12 (hereafter referred to as B04 and B12) demonstrated a 5.8- and 650-fold faster relative off-rate at pH 5.5 than at pH 7.4 in this setup.

**Table 1:** Affinities and off-rates of anti-M-II and anti-α-cbtx IgG1 variants

Antigen	IgG1	K_D_ at pH 7.4 (M)	k_off_ (s^−1^)		k_off_ fold difference (pH 5.5/7.4)
			pH 7.4	pH 5.5	
M-II	TPL0039_05_A03^44^	<1 x 10^−12^	<1.0 x 10^−7^	<1.0 x 10^−7^	ND
	TPL0039_05_B04^44^	75 x 10^−12^	6.5 x 10^−5^	3.8 x 10^−4^	5.8
	TPL0039_05_B12^44^	<1 x 10^−12^	<1.0 x 10^−7^	6.5 x 10^−5^	650
	TPL0039_05_E02^44^	<1 x 10^−12^	<1.0 x 10^−7^	<1.0 x 10^−7^	ND
α-cbtx	TPL0197_01_C08	3.2 x 10^−9^	1.3 x 10^−4^	2.8 x 10^−4^	2.1

Binding constants (K_D_) of anti-M-II and anti-α-cbtx targeting IgG1s at pH 7.4, their off-rates (k_off_) at pH 7.4 and 5.5, and the fold difference between the off-rates at the two pH values. A k_off_ of <1·10^−7^ s^−1^ implies that the IgG1 did not show any dissociation from the antigen in the observed dissociation period of 300 s. ND stands for not determined.

**Figure 1:**
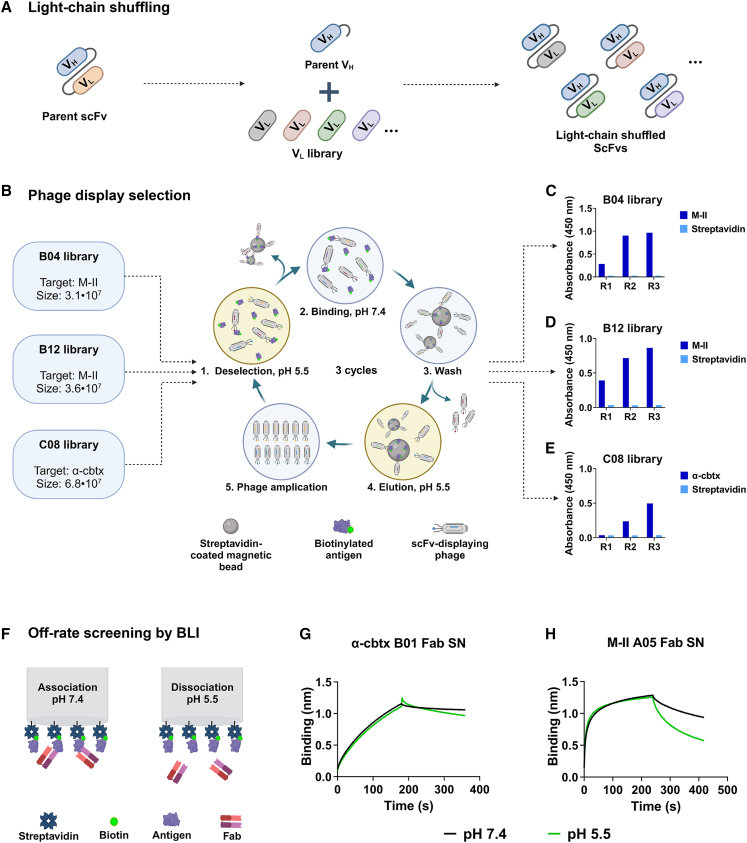
Phage display selection and binding of reformatted Fab fragments to snake toxins (A–E) Schematic illustration of the (A) light-chain shuffling and (B) phage display selection campaigns for isolation of scFvs with pH-dependent antigen binding properties derived from the B04, B12, and C08 light-chain shuffled libraries. (1) scFv-displaying phages were incubated with biotinylated antigen at pH 5.5, which was followed by capture and removal of phages that bound the antigen at low pH using streptavidin-coated magnetic beads. (2) The phages that did not bind to the antigen at pH 5.5 were collected and incubated with biotinylated antigen at pH 7.4 prior to capture on streptavidin-coated magnetic beads. (3) The beads were washed to remove unspecific phages. (4) The bound phages were then eluted using a pH 5.5 buffer and (5) amplified for the next round of selection. Three consecutive rounds of selection were performed with the generated libraries. Polyclonal phage ELISA of the phage outputs from the three rounds of selection performed with libraries (C) B04, (D) B12, and (E) C08 showing that the outputs bind to their respective cognate antigens and that minimal binding to streptavidin is detected. (F–H) (F) Schematic illustration of the off-rate screening approach using BLI, where the association was performed at pH 7.4, which was followed by dissociation for 300 s at pH 7.4 or pH 5.5. BLI sensorgrams of Fab-containing supernatant from CHO cell expression experiments of (G) B01 and (H) A05 showing binding to α-cbtx and M-II, respectively, at pH 7.4, followed by dissociation at pH 7.4 or 5.5. (SN, supernatant).

To investigate if light-chain shuffling could be used to engineer the IgG1s to exhibit more pronounced pH-dependent antigen binding properties, the variable heavy chains of B04 and B12 were employed and combined with a variety of variable light chains to generate light-chain shuffled scFv phage display libraries^51^ (Figure 1A). This yielded libraries with a clonal diversity of 3.1·10^7^ and 3.6·10^7^ for B04 and B12, respectively. In addition, we light-chain shuffled a previously discovered anti-α-cbtx IgG1, TPL0197_01_C08^38^ (referred to as C08) with pH-dependent antigen binding properties, which showed a 2-fold faster off-rate at pH 5.5 than at neutral pH (Table 1). The clonal diversity of the C08 library was determined to be 6.8·10^7^.

To select scFvs with improved pH-dependent antigen binding properties, the C08 library was panned against α-cbtx, and the B04 and B12 libraries were panned against M-II (Figure 1B). The panning rounds were performed with a deselection step where phages binding biotinylated antigens at low pH (pH ∼ 5.5) were removed before panning on biotinylated antigens at pH 7.4, and elution was performed using low pH (Figure 1B). An ELISA was then performed to assess the polyclonal phage outputs, which showed enrichment of antigen-specific scFvs and negligible binding to the negative control (streptavidin) (Figures 1C–1E). The scFv-encoding cDNA sequences from the third selection round from each library were then subcloned for expression of soluble scFvs in *E. coli*.

In total, 184 scFvs from each library were screened for binding in an expression-normalized capture dissociation-enhanced lanthanide immunoassay (DELFIA), which was used to rank the scFvs based on target binding at pH 7.4. Binders were defined by having signals above a threshold value of 10,000 (10 times above the background). From the C08 library, 23 scFvs were shown to bind α-cbtx, while the numbers of M-II binders were 52 for the B04 library and 104 for the B12 library (Figures S1A–S1C). Subsequently, 10 α-cbtx-binding clones from the C08 library and 33 and 49 M-II-binding clones from the B04 and B12 libraries, respectively, were sequenced. The sequencing revealed 1 unique α-cbtx-binding scFv, 12 unique M-II-binding scFvs from the B04 library, and 20 unique M-II-binding scFvs from the B12 library.

Next, the V_H_ and V_L_ genes from the scFv clones were reformatted to Fabs and transiently expressed in Chinese hamster ovary (CHO) cells. The Fab-containing cell supernatant was employed to perform an off-rate screening against the toxins at pH 7.4 and 5.5 using BLI (Figure 1F). Surprisingly, the α-cbtx-targeting Fab, TPL0544_01_B01 (hereafter referred to as B01), showed a similar off-rate at both pH conditions (Figure 1G), while the M-II-targeting Fab, TPL0552_02_A05 (hereafter referred to as A05), from the B04 library showed a faster off-rate at pH 5.5 than pH 7.4 (Figure 1H). All other Fabs from the B04 and B12 libraries were not affected by pH alteration.

### pH-dependent antigen binding properties of A05 confirmed by off-rate screening through a pH gradient

To further investigate the effect of pH on the antigen binding properties of the discovered antibodies, we determined the off-rates for B01 and A05 across a pH gradient from 7.5 to 3.5 using BLI. For comparison, the parental C08 and B04 Fabs and an anti-M-II A03 Fab binding equally well at both pH 5. 5 and 7.4 (Table 1) were included. In addition, two previously discovered α-cbtx-targeting Fabs, A01 and D11,^45^^,^^52^ which coincidentally displayed pH-dependent antigen binding and non-pH-dependent antigen binding properties, respectively, were included as positive and negative controls.

**Figure 2:**
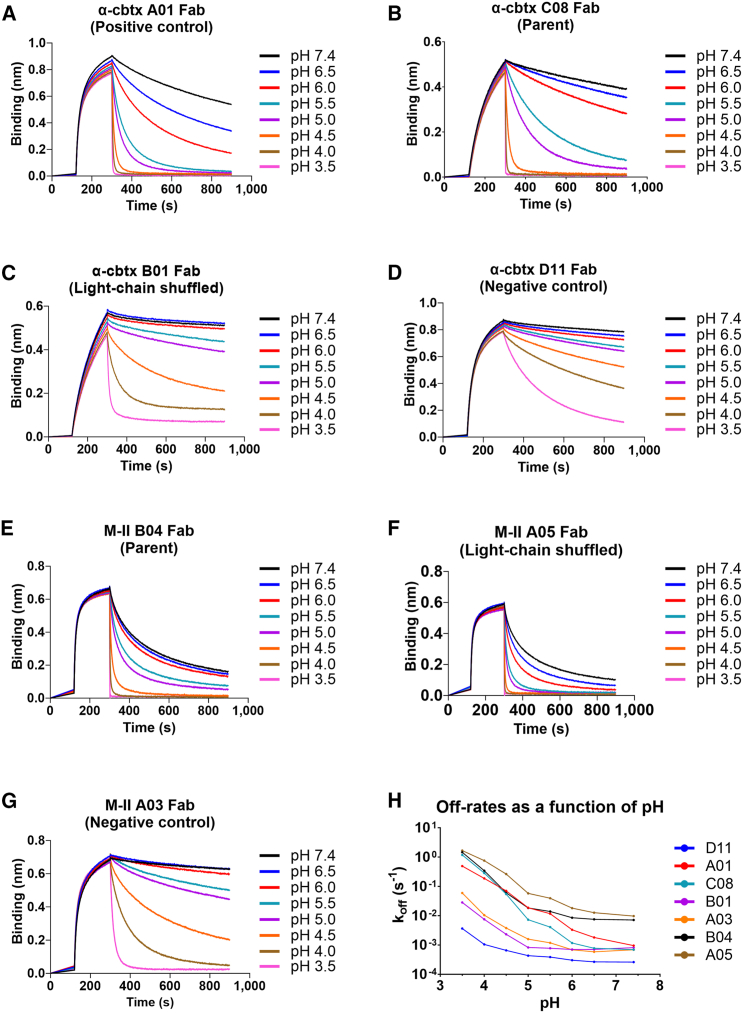
Dissociation of the Fabs measured through a pH gradient using BLI (A–G) Sensorgrams for the α-cbtx-targeting Fabs: (A) Fab A01 (positive control for pH-dependent antigen binding properties), (B) parental Fab C08, (C) light-chain shuffled Fab B01, and (D) Fab D11 (negative control with non-pH-dependent antigen binding properties). Sensorgrams for the M-II-targeting Fabs: (E) Parental Fab B04, (F) light-chain shuffled Fab A05, and (G) Fab A03 (negative control with non-pH-dependent antigen binding properties). Off-rates were determined at pH 3.5, 4.0, 4.5, 5.0, 5.5, 6.0, 6.5, and 7.4. The negative Fab controls showed similar off-rates at pH 5.5 and 7.4. (H) pH versus off-rate (k_off_) plot for the assessed Fabs.

As expected, the off-rate of all Fabs from the toxin-loaded biosensor increased with decreasing pH (Figure 2; Table S1). Specifically, while Fab A01 and the parent Fab C08 showed increasing off-rates at pH 5.5 compared to pH 7.4 (Figures 2A, 2B, and 2H), a faster dissociation was measured for the light-chain shuffled Fab B01 at pH 4.5 and below (Figures 2C and 2H). In comparison, Fab D11 showed slower off-rates over the pH range compared with the other Fabs (Figures 2D and 2H).

In the case of the M-II-targeting mAbs, the light-chain shuffled Fab A05 showed faster off-rates with decreasing pH than the parent Fab B04 (Figures 2E and 2F). In comparison, Fab A03 showed a slower off-rate at pH 7.4, with a pH-dependent off-rate profile that was very similar to the α-cbtx-targeting Fab B01 (Figures 2G and 2H). Additionally, the K_D_s of the Fabs were determined at pH 7.4 and pH 5.5 using BLI (Table S2). This revealed that the α-cbtx-targeting parent Fab C08 and the light-chain shuffled Fab B01 showed a similar K_D_ ∼ 50 nM at pH 7.4, while, at pH 5.5, the parent C08 showed a 7.5-fold higher K_D_ (1.5 μM) than the light-chain shuffled Fab B01 (200 nM). In the case of the M-II-targeting parent Fab B04 and the light-chain shuffled Fab A05, their K_D_s were ∼20 nM at pH 7.4. However, at pH 5.5, the parent B04 showed a 3-fold lower K_D_ (77 nM) than the light-chain shuffled clone A05 (230 nM).

### Sequence analysis reveals the presence of a histidine residue in the variable light chain of A05

To compare the variable sequence differences between the light-chain shuffled A05 and B01 and the corresponding parental clones B04 and C08, respectively, sequence alignments and comparisons to germline were made. The results revealed that the light-chain shuffled versions showed an identical or similar identity to the germline as the parental antibodies (Table S3). Furthermore, the alignment showed that A05 had gained 6 amino acid changes compared to the parent B04, in which a tyrosine residue in the light-chain complementarity-determining region 3 (CDR-L3) of B04 was replaced with a histidine residue in A05 (Figure S2A), which might explain its pH-dependent antigen binding properties. In the case of B01, which showed similar off-rates at pH 5.5 and 7.4, the analysis revealed an astonishing 66 amino acid changes compared to the parent C08, including an asparagine residue in the framework region 2 that was replaced with a histidine residue (Figure S2B).

To structurally characterize the influence of protonation on antigen binding, we performed molecular dynamics simulations of C08 and B01 in complex with α-cbtx and B04 and A05 in complex with M-II at pH 7.5 and pH 5.5 and investigated the interaction energies (Figure S3). The interaction energies reveal substantial variations between different protonation states with a drastic increase in interaction energies at lower pH values. Smaller changes in interaction energies could be identified for B01 and B04. For the M-II-targeting mAbs, the histidine residue in the CDR-L3 that differs between B04 and A05 (Y91H) gets protonated at lower pH values. This protonation allows more water molecules to enter the binding interface, thereby destabilizing the complex, which may explain the increased pH-dependent antigen binding properties of A05. The increase in water interactions of this histidine residue at pH 5.5 is confirmed by a substantial decrease in water interaction energy from −11 to −53 kcal/mol, which is accompanied by a higher variability of the A05-M-II complex (resulting in a higher number of clusters at lower pH ranges, i.e., 12 clusters at pH 7.5 and 34 clusters at pH 5.5).

For the α-cbtx-targeting mAbs, C08 does not contain any histidine residues in the Fv. However, the interplay between water and the glutamate residues situated in the V_H_-V_L_ interface between CDR-L2 (55E) and CDR-H3 (95E and 96E) results in a higher interaction energy at lower pH values, potentially explaining the observed faster off-rates at lower pH for this antibody (Figure S3). While B01 differs substantially in sequence from the parental C08, the predicted antibody-antigen complex shows that the CDR-H3 loop remains the main interaction site for α-cbtx in both antibodies, and the two adjacent glutamate residues in the CDR-H3 loop (95E and 96E), in combination with the N34H mutation, seem to be critical for the pH-dependent antigen binding properties of B01.

To evaluate the thermal stability of the parental and light-chain shuffled clones, the melting temperatures, T_M_, were analyzed using differential scanning fluorimetry. This showed that the T_M_ for C08 versus B01 were 67.5°C and 66.1°C, respectively, and 76.5°C and 75.2°C for B04 versus A05, respectively (Figure S4), indicating a similar stability for the light-chain shuffled clones compared to the parental ones.

### Fc-engineering for enhanced binding to hFcRn

To study the hFcRn binding properties of the light-chain shuffled A05 and B01, seven different clones were transiently expressed as full-length IgG1 mAbs in CHO cells. The IgG1s were expressed with the YTE amino acid substitutions to combine the effects of pH-dependent antigen binding properties with Fc-engineering for enhanced hFcRn binding and the LALA amino acid substitutions to reduce binding of effector molecules (here, IgG1-YTE refers to IgGs with all substitutions). Additionally, D11 and A03 were made with WT Fcs. An overview of the designed IgG1 variants is given in Table 2. All full-length IgG1 mAbs were determined to be monomeric using analytical size-exclusion chromatography (SEC) (Figure S5).

**Table 2:** Release from hFcRn as a function of pH

Target	IgG1	Fc	Elution peak pH	
			IgG1	IgG1-antigen complex
α-cbtx	2554_01_D11	WT	6.95	6.84
	2554_01_D11	YTE	7.55	7.47
	2555_01_A01	YTE	7.56	7.54
	TPL0544_01_B01	YTE	7.57	7.50
	TPL0197_01_C08	YTE	7.53	7.51
M-II	TPL0039_05_A03	WT	7.21	7.30
	TPL0039_05_A03	YTE	7.73	7.88/8.12^a^
	TPL0039_05_B04	YTE	7.59	7.59
	TPL0552_02_A05	YTE	7.55	7.55

Elution peak pH values for IgG1 variants alone and in complex with their cognate antigens following release from the hFcRn-coupled column. The IgG1s were incubated with the antigens at a 1:2 molar ratio.

a Denotes double elution peaks.

To validate the BLI data, the produced anti-α-cbtx IgG1s were analyzed using surface plasmon resonance (SPR) with dissociation at pH 5.5 and pH 7.4. The IgG1s retained their binding to α-cbtx at pH 7.4, with very little dissociation occurring within the first 300 s. In contrast, at pH 5.5, a faster dissociation was observed for C08 and A01, confirming that these clones indeed possess pH-dependent antigen binding properties (Figure S6). In addition, single-cycle kinetics experiments were performed, which showed that the IgG1s displayed similar binding kinetics (Table S4) as observed earlier with the Fabs (Table S2).

**Figure 3:**
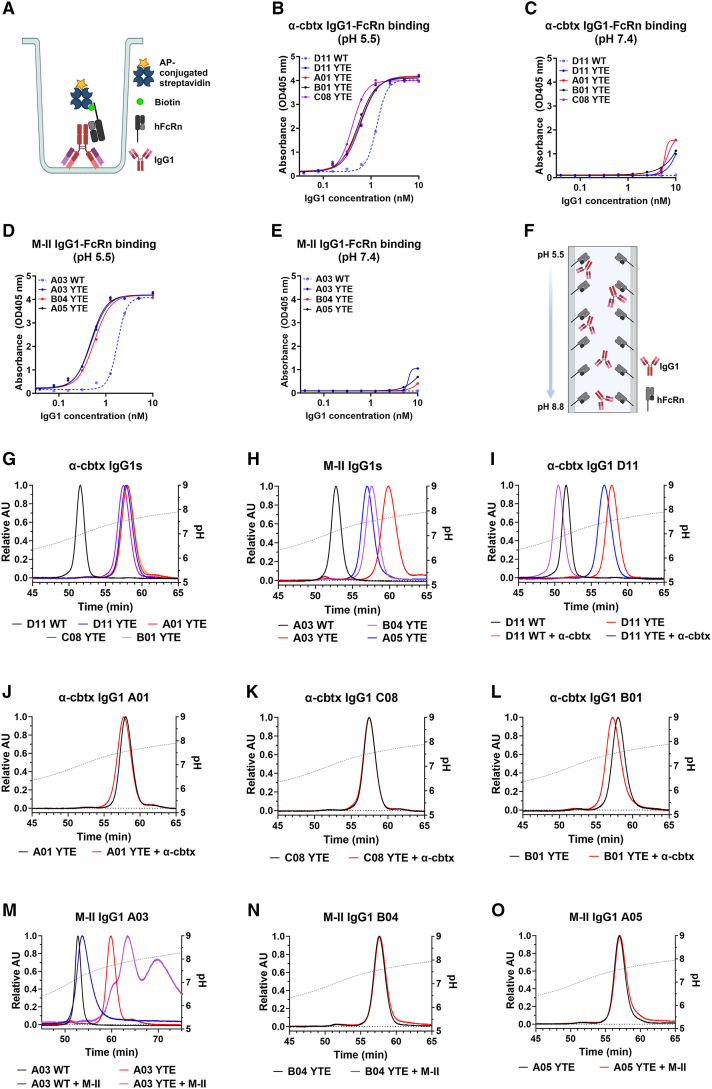
The engineered IgG1 variants show distinct hFcRn binding properties in the absence and presence of their antigens (A–E) (A) Schematic illustration of the ELISA setup used to detect binding between the IgG1 variants and biotinylated hFcRn. Binding of biotinylated hFcRn to WT and YTE-containing IgG1 variants targeting α-cbtx (B and C) and M-II (D and E) at pH 5.5 and pH 7.4. Data shown as mean ± SD of duplicates. (F–O) (F) Schematic illustration of the hFcRn affinity chromatography experiment, where the pH varies from 5.5 to 8.8, used to assess the release of IgG1s from the receptor. Elution profiles of WT and YTE-containing IgG1 variants targeting (G) α-cbtx and (H) M-II from the hFcRn-coupled column are shown as relative absorbance units as a function of a pH gradient. Elution profiles of WT and YTE-containing the IgG1 variants when pre-incubated with (I–L) α-cbtx or (M–O) M-II at pH 5.5 are shown as the relative absorbance as a function of pH. The pH is plotted on the right Y axis (dotted line). Figures (A) and (F) were created with BioRender.

To evaluate the ability of the IgG1s to bind hFcRn, an ELISA was performed (Figure 3A). This revealed that all IgG1 variants bound the receptor at pH 5.5 (Figures 3B and 3D), with increased binding of the YTE-containing variants, while only residual (YTE) or no binding (WT) of the IgG1s was observed at neutral pH (Figures 3C and 3E).

To mimic the pH-dependent binding and release that take place in the endosomal pathway, we used an hFcRn-coupled column to study how the IgG1s behaved when they were injected at pH 5.5 and subjected to a gradual pH increase to pH 8.8^53^ (Figure 3F). The generated elution profiles revealed that the YTE-containing variants were retained for a longer time on the column and, as such, released from the receptor at a higher pH than the WT counterparts (Figures 3G and 3H; Table 2). For the anti-α-cbtx IgG1s, the four YTE-containing variants were shown to elute at a consistent pH of 7.5, which was notably higher than that for the WT counterpart, which eluted at pH 6.8 (Figures 3G; Table 2). More variation in elution profiles was observed for the anti-M-II IgG1-YTE variants, where A03 was retained on the column the longest (pH 7.7), followed by B04 (pH 7.59) and A05 (pH 7.55) (Figures 3H; Table 2). As described previously, the observed pH discrepancies in antibody elution may be partially attributed to variations in the sequences of the variable regions.^20^^,^^54^ Specifically, the antibody framework of the A03 IgG exhibits a notably higher positive charge compared to the frameworks for the other anti-M-II antibodies when comparing the YTE variants (Figure S7). Furthermore, a striking difference was detected for the two WT D11 and A03 variants, as anti-M-II A03 was eluted at pH 7.21 compared with pH 6.95 for anti-α-cbtx D11 (Table 2). Thus, these results strongly indicate that the variable region differences of the IgG1 mAbs modulate the release from hFcRn, while Fc-engineering for improved hFcRn engagement at low pH leads to a delay in the release from the receptor.

### Engagement of hFcRn is influenced by antigen binding

Each IgG1 molecule has two antigen-binding sites; M-II exists in both monomeric and oligomeric forms in solution,^55^ and α-cbtx exists in monomeric and dimeric forms.^56^ As such, the difference in the number of binding sites per antigen may affect the size of the IgG1-antigen complexes formed upon mixing the IgG1s with their cognate toxins. To evaluate the sizes of the complexes formed, IgG1s and antigens were mixed at a 1:20 molar ratio and studied using mass photometry. For all IgG1s, it was found that addition of antigen increases the molecular mass of the dominant species by a mass twice that of the antigen, thus indicating a simple 1:2 binding (Figure S8). The B04 antibody also showed the formation of larger complexes with molecular masses of 400 and 600 kDa upon addition of M-II, though at a much lower concentration (∼1%) than the dominant species (Figure S8C). This complex formation is expected to be dependent on the antibody concentration.

To investigate how the formation of IgG1-antigen complexes affects binding to hFcRn, each complex at a 1:2 molar ratio between the IgG1 and its cognate antigen was applied to the receptor-coupled column (Figures 3I–3O). The results revealed that both the WT and YTE variants of IgG1 D11 eluted earlier in the presence of α-cbtx, at pH 6.84 and 7.47, respectively, than in the absence of antigen (pH 6.95 and 7.55) (Figure 3I). In contrast, the YTE-containing A01 and C08 variants showed no or only minor changes in retention time in the presence of α-cbtx, while B01-YTE eluted earlier (pH 7.50) when α-cbtx was present (Figures 3J–3l). Similarly, no effect of the M-II antigen was measured for IgG1 B04-YTE and A05-YTE (Figures 3N and 3O), while the WT and YTE-containing variants of IgG1 A03 were shown to be retained on the column for a longer time, in which A03-YTE stayed bound the longest and gave rise to two elution peaks (Figure 3M) when incubated with the M-II antigen. Thus, release from hFcRn as a function of pH is affected by the composition of each IgG1 and the two distinct cognate antigens.

### Antigen binding alters the cellular transport properties of IgG1 variants

**Figure 4:**
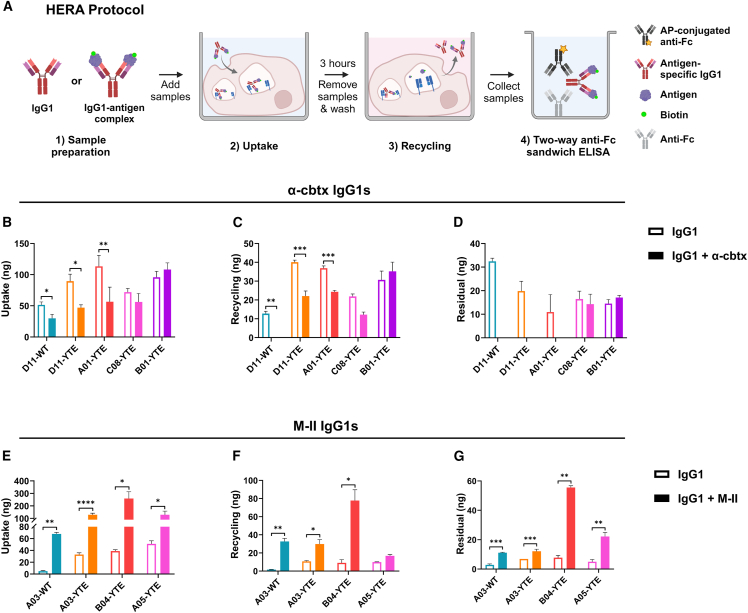
Cellular transport properties of the IgG1 variants in the absence and presence of antigen (A) Schematic overview of the HERA protocol. The IgG1 variants were pre-incubated with or without their cognate antigen and added to the hFcRn-expressing HMEC-1 cells (1). After incubation for 3 h to allow for cellular uptake of the IgG1s (2), the medium was removed, and the cells were washed prior to lysis and collection of samples. In parallel, cells were washed, and fresh medium was added, which was followed by a 3-h incubation step to allow recycling and release into the medium. Medium was then collected, and cells were lysed (3). A two-way anti-Fc ELISA was used to measure the presence of IgGs in the collected medium and lysate samples (4). The figure was created with BioRender. ELISA quantification of the amounts of IgG1 taken up, recycled, or accumulated in the absence and presence of cognate antigen for (B–D) anti-α-cbtx IgG1 and (E–G) anti-M-II IgG1 variants. Data shown as mean ± SD of one representative experiment with triplicates (*n* = 3 per data point). *^∗^p* > 0.05, *^∗∗^p* > 0.01, *^∗∗∗^p* > 0.001, *^∗∗∗∗^p* > 0.0001 (unpaired Student’s t test).

To address how the differences in hFcRn engagement in the absence and presence of antigen translated into cellular transport properties, we took advantage of a human endothelial cell-based recycling assay (HERA).^53^ This assay is based on a cell line (HMEC-1) that over-expresses hFcRn and can be used to measure cellular uptake and receptor-dependent rescue from intracellular degradation. The cells were exposed to the IgG1s followed by incubation to allow for uptake, whereafter the cells were washed and either lysed or incubated with growth medium depleted of IgG. After an additional 3 h of incubation, the medium was collected, and the cells were washed and lysed (Figure 4A). Using a two-way anti-Fc ELISA to quantify the presence of IgG1 in the collected samples, about 2- to 10-fold more of the YTE-containing IgG1 variants could be detected inside the cells after the uptake phase, and 2- to 7-fold more of these variants could be detected in the medium following the recycling step compared with the WT counterparts (Figures 4B, 4C, 4E, and 4F). The only exception was the anti-α-cbtx YTE-containing IgG1 C08, which showed an unexpectedly similar phenotype as the D11-WT (Figures 4B and 4C). Regarding the detected residual amounts, the anti-α-cbtx IgGs containing YTE substitutions showed 1.5- to 3-fold lower accumulation inside the cells. However this phenomenon was not observed for the anti-M-II IgGs (Figures 4D and 4G).

To address if and how the cellular transport properties of the IgGs were affected by the presence of cognate antigens, the IgGs were pre-incubated with their antigens in a 1:2 molar ratio before addition to the cells (Figure 4A). HERA was then repeated as before, which revealed a 1.7-fold reduced uptake of WT D11 and no detection of recycling and residual sample inside the cell (Figures 4B–4D). The same phenotype was measured for the YTE-containing D11 and A01, while transport of anti-α-cbtx C08-YTE and B01-YTE was unaffected by the presence of antigen (Figures 4B–4D). On the contrary, when the anti-M-II WT IgG1 A03 was pre-incubated with its antigen, an increase in uptake of about 13-fold was measured, with a similarly enhanced ability to be recycled and a significant increase in residual amounts (Figures 4E–4G). All the anti-M-II YTE-containing variants displayed a similar phenotype, for which neither the uptake nor the recycling of YTE-containing IgG1s in complex with M-II was enhanced beyond that of the WT IgG1 A03-M-II complex (Figures 4E–4G).

### IgG1 variants with pH-dependent antigen binding properties are recycled without their antigen

**Figure 5:**
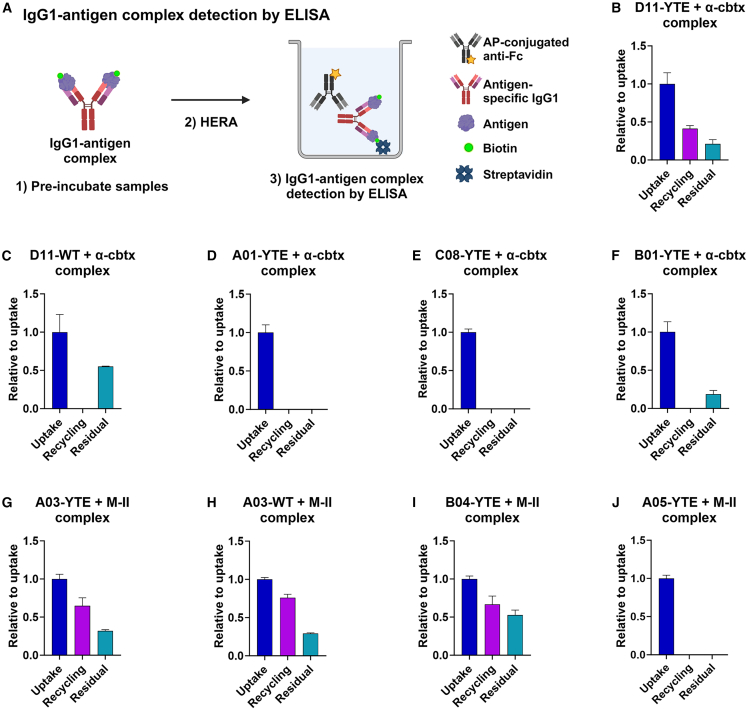
Tracking of antigen-bound IgG1 variants following cellular transport in HERA (A) Schematic overview of the protocol for measuring IgG1 variants bound to their cognate antigen during cellular uptake, recycling, and termination of HERA. (1) IgG1 variants were pre-incubated with or without biotinylated antigens and (2) analyzed with HERA. (3) IgG1s present in the collected lysates and recycling media were quantified by an ELISA, where biotinylated antigens were captured on streptavidin-coated wells followed by detection of bound IgG1 with an anti-Fc antibody. The figure was created with BioRender. ELISA results showing the uptake, recycling, and accumulation of antigen-bound (B–F) anti-α-cbtx IgG1 and (G–J) anti-M-II IgG1 variants. For each IgG1-antigen complex, the relative uptake, recycling, and residue amounts were normalized to the obtained uptake value of the complex. Data shown as mean ± SD of one representative experiment with triplicates (*n* = 3 per data point).

To investigate if the IgG1s were recycled with (bound) or without (unbound) their cognate antigens, we established an ELISA in which biotinylated antigens were captured on streptavidin-coated wells, and where bound IgG1 was detected with an anti-Fc antibody (Figure 5A). Screening of the HERA samples showed that both the anti-α-cbtx and anti-M-II IgG1 variants were bound to their antigens after cellular uptake (Figures 5B–5J). Comparing the anti-α-cbtx IgG1s revealed that only D11-YTE was detected bound to its antigen following recycling (Figure 5B), which was not the case for the WT counterpart, confirming the non-detectable recycling of antigen-bound WT IgG1 D11 detected using the two-way anti-Fc ELISA (Figures 4C and 5C). Regarding the positive control IgG1 with pH-dependent antigen binding properties, A01-YTE, and the parental clone, C08-YTE, none of these were detected as complexes in either of the recycled or residual samples (Figures 5D and 5E). Similar results were obtained for the light-chain shuffled IgG1 B01-YTE, although some cellular accumulation of the complex was measured (Figure 5F). The absence of IgG1 B01-YTE in complex with its antigen in the recycled sample was surprising, since B01 showed a 6- and 16-fold slower off-rate at pH 5.5 than C08 and A01, respectively (Figure 2H).

Regarding the anti-M-II IgG1 variants, both the WT and YTE versions of A03 together with B04-YTE were recycled in complex with M-II or accumulated inside the cells (Figures 5G–5I). This was not the case for the light-chain shuffled A05-YTE that was not detected in complex with M-II in the recycled or residual samples (Figure 5J), in line with its pH-dependent mode of antigen binding (Figure 2F). Thus, the pH-dependent antigen binding properties of IgG1 A01, C08, B01, and A05 were confirmed in the cellular experiments.

## Discussion

The prevalent approach for developing acid-switched mAbs involves the introduction of histidine residues in the CDRs of the mAbs. However, this strategy carries the risk of compromising antibody-antigen binding at neutral pH^13^^,^^28^ and introducing sequence liabilities that could lead to issues related to developability and immunogenicity.^57^ While these challenges may also exist for *in vitro*-discovered mAbs with non-native pairing of the V_H_ and V_L_ domains, further artificial introduction of histidine residues in the CDRs may be undesirable, and complementary approaches for introducing pH-dependent antigen binding properties are therefore warranted.

Here, we demonstrate that light-chain shuffling coupled with phage display selection can serve as a useful tool to generate or enhance pH-dependent antigen binding properties of IgG1s against soluble targets without observing any unspecific binding or altering the native sequences of the variable domains (i.e., without artificially introducing histidine residues in the CDRs). This emphasizes the important role of the light chain for modulating antigen binding properties by determining the shape and conformational diversity of the heavy-chain CDR loops, in particular the CDR-H3 loop.^58^^,^^59^ We speculate that this approach can be more generally used to develop mAbs with V_H_ and V_L_ pairings that show increased pH-dependent antigen binding properties through mechanisms such as (1) increased solvent accessibility for amino acid residues that can accept or donate protons (possibly in the V_H_ and V_L_ interface), (2) alteration in the intramolecular hydrogen bonding network, and (3) selection of light chains with a naturally high histidine residue content, or combinations thereof. In alignment with previous studies, we also demonstrate that hFcRn binding and cellular uptake and recycling are not only determined by the constant Fc region of the IgG but also modulated by the composition of their variable regions,^54^^,^^60^^,^^61^^,^^62^ binding to the cognate antigen,^19^^,^^54^ and the size of the antibody-antigen complex in question.^22^ This is, for example, illustrated by the light-chain shuffled IgG1 B01 that contains a lambda light chain, which exhibits higher cellular uptake and recycling than the kappa light chain containing parental clone IgG1 C08 (Figures 4B and 4C). This also illustrates that the two different light chains influence the engagement of hFcRn and the cellular transport properties of the IgG1. In relation to the influence of bound antigen on the IgG1-FcRn interaction and the cellular recycling of the antibody, we showed that IgG1 D11 and B01 elute from the hFcRn column at lower pH when bound to α-cbtx than the unbound IgG1s (Figures 3I and 3L), while this was not the case for IgG1 A01 and C08 (Figures 3J and 3K). This could be due to the fast dissociation rates and low affinity between α-cbtx and IgG1 A01 and C08 at pH 5.5, resulting in a majority of the IgG1 being unbound during the process. In the HERA experiments, we measured a reduction in the uptake and recycling of IgG1s D11 and A01 in the presence of α-cbtx, which was not observed for IgG1s C08 and B01 (Figures 4B and 4C), while all tested anti-M-II-targeting IgG1s showed increased uptake and recycling in the presence of M-II (Figures 4E and 4F). As M-II exerts its function by interacting with plasma membranes,^47^ these observations could potentially be explained by hypothetical toxin-mediated enhancement mechanisms that increase the cellular uptake by bringing the IgG1-antigen complexes close to the cell membrane where the complexes can engage with hFcRn.^44^^,^^49^ Importantly, the reduced uptake of the acid-switched IgG1 A01 in the presence of α-cbtx could affect its function *in vivo* by possibly extending the antigens’ half-life in circulation.^32^ Thus, to ensure optimal cellular uptake and recycling properties of acid-switched IgG1s, it is crucial to take into account the biophysical attributes of the variable regions, the biochemistry and mode of action of the cognate antigens,^32^ and the cellular handling characteristics of the IgG1-antigen complexes and compare these properties with those of unbound IgG1 counterparts.

Furthermore, the pH-dependent antigen binding properties of the IgG1 A01 (positive control with pH-dependent antigen binding properties), C08 (parent), and B01 and A05 (light-chain shuffled IgG1s) were studied in HERA. The results revealed that IgG1s B01 and A03 possessed different cellular transport properties. Specifically, no α-cbtx-B01 complexes were observed in the recycling sample compared to the samples containing M-II-A03 complexes, for which a high level of recycled complexes was detected (Figures 5F and 5G), even though both mAbs exhibited similar relative off-rate profiles at pH 3.5–7.4 (Figure 2H). This difference in their cellular transport properties can potentially be explained by the more than 30 times higher affinity of A03 toward M-II (K_D_ ∼ 6 nM) than that of B01 toward α-cbtx (K_D_ ∼ 209 nM). Considering overall affinity, rather than solely focusing on dissociation rates, may thus be relevant when assessing the ability of an antibody to engage with its target antigen in a pH-dependent manner.

Interestingly, analytical hFcRn chromatography revealed distinct retention times of A03 compared with the other M-II-binding mAbs. While most IgG1-antigen complexes displayed no or minor shifts to later retention times compared to the antibody alone, the behavior of A03 was different (Figure 3M). For the mAb alone, A03-YTE displayed a longer retention time compared to the WT IgG (60 vs. 52 min), and, upon preincubation with the antigen, A03-YTE eluted at 63 min and 70 min in the pH gradient. Usually, such a phenomenon can sometimes be observed with more high-affinity interactions like the YTE-Fc binding to FcRn and is a result of an avidity effect after target binding.^63^ This may be reasoned by antigen oligomerization in the presence of bivalent IgG1 mAbs presenting more than one Fc to the column-coupled hFcRn. However, mass photometry indicated that complexes with sizes equivalent to a 1:2 binding ratio between IgG and antigen were present at the tested concentration for all included mAbs, with only a small fraction of larger complexes being present for B04 (Figure S8C). Neither B04 nor A05 showed any late eluting species. Therefore, the longer retention time for A03-YTE may instead relate to a faster dissociation from M-II or an overall lower affinity. Together, this highlights the complexity of predicting the FcRn binding and recycling properties of antibodies.

So far, acid-switched IgG1s have primarily been studied for their utility in targeting endogenous antigens that are continuously generated in the body, where instant neutralization is typically not crucial.^13^^,^^23^^,^^25^^,^^26^^,^^27^ Here, we focused on using the snake venom toxins, α-cbtx and M-II, as target antigens, which are of exogenous origin to mammals. Upon envenoming, these toxins are instantly delivered in substantial quantities at the bite site^37^ and may be released from the bite site into the bloodstream over time (depot effect). Therefore, the current treatment for snakebite envenoming, reliant on antivenoms based on polyclonal antibodies derived from immunized animals, requires exceptionally high doses to effectively neutralize the venom toxins.^64^ Thus, the use of acid-switched IgG1s may present an attractive strategy for the development of antibody cocktails (i.e., recombinant antivenoms) that can neutralize snake venoms at lower doses compared to antivenoms based on antibodies with non-pH-dependent antigen binding properties, such as the commonly used polyclonal, heterologous Fab and F(ab’)_2_-based antivenoms.^38^ In turn, this might potentially lead to a lower cost of treatment, which is key for the deployment of antivenoms for snakebite envenoming therapy in the rural tropics.^37^^,^^65^

As the *in vivo* properties of the discovered acid-switched antibodies have not yet been thoroughly assessed, it is unknown how effectively such antibodies would eliminate the antigens, especially in circumstances involving complex toxicokinetics. In this regard, a recent study using the parental B12 mAb showed that this IgG1 possessed potent *in vivo* neutralizing capabilities against the myotoxic effects of M-II and *B. asper* whole venom when it was assessed in CD-1 mice by intramuscular injection of a pre-incubated mixture of toxin or venom and B12.^44^ However, upon switching from preincubating the venom and mAb before intramuscular injection to first injecting the venom intramuscularly and then injecting B12 intravenously after a time delay (i.e., a rescue assay), the mAb instead increased the toxicity of the venom.^44^ This antibody-dependent enhancement of toxicity (ADET) was shown to potentially be related to the YTE Fc-engineering,^15^ as ADET was not observed for the WT B12 counterpart. While the authors suggest a potential link between the observed ADET and the modified recycling properties of the mAb due to YTE,^44^ it should be noted that such Fc-engineering has been shown to cause endosomal human IgG1 accumulation and reduced plasma half-life compared to WT IgG1 in mice expressing endogenous FcRn due to altered pH-dependent binding kinetics.^15^^,^^53^ Based on this, we speculate that the presence of B12-YTE in such mice challenged with M-II may lead to an increased endosomal accumulation of antibody-M-II complexes due to poor FcRn-mediated recycling, where the toxin, bound or unbound to the mAb, may exert increased toxicity. Thus, the enhanced toxicity could be an effect of cross-species binding differences. Therefore, conducting similar rescue assays in transgenic mice expressing the human form of FcRn might be beneficial to elucidate the mechanisms behind the observed ADET.

Moreover, we further contemplate that the utility of acid-switched IgG antibodies and half-life extension strategies may depend on the mode of action as well as the molecular properties of the target antigen(s) to be neutralized—both in the field of toxinology and beyond. In relation to the neutralization of snake toxins, it would be attractive to conduct *in vivo* efficacy studies on the discovered acid-switched (human) IgG1 antibodies in mice reflecting hFcRn biology.^66^ This is essential, as novel snakebite therapeutics must be evaluated in relevant preclinical models to gain a better understanding of their utility in a translational perspective.^67^

Despite the aforementioned limitations, the methodologies employed in this study can be more generally applied to discover and/or improve acid-switched IgG mAbs against distinct targets, as well as to assess the pH-dependent antigen binding properties of antibodies in a cellular setting, taking hFcRn biology into consideration. For instance, the methods might be attractive for the generation of efficacious acid-switched antibody-based therapies against infectious diseases, which are often characterized by the release of large amounts of harmful toxins and virulence factors. Perhaps, even more importantly, the benefit that our methods preserve the human sequences of the mAbs could also be of great value when targeting endogenous antigens that drive chronic diseases, such as in autoimmunity, as the use of fully human mAbs may come with a lower risk of inducing anti-drug antibodies when the mAb is used frequently over time.^68^^,^^69^

## STAR★Methods

### Key resources table

**Table:** 

REAGENT or RESOURCE	SOURCE	IDENTIFIER
**Antibodies**		

anti-M13 horseradish peroxidase	Sino Biological	11973-MM05T-H; RRID: AB_2857928
IgG: TPL0197_01_C08-YTE	Tulika et al.^38^	N/A
IgG: TPL0039_05_A03-YTE	Sørensen et al.^44^	N/A
IgG: TPL0039_05_E02-YTE	Sørensen et al.^44^	N/A
IgG: TPL0039_05_B04-YTE	Sørensen et al.^44^	N/A
IgG: TPL0039_05_B12-YTE	Sørensen et al.^44^	N/A
IgG: TPL0038_05_A03-WT	This paper	N/A
IgG: TPL0544 _01_B01-YTE	This paper	N/A
IgG: TPL0552_01_A05-YTE	This paper	N/A
IgG: 2555_01_A01-YTE	This paper	N/A
IgG: 2555_01_A01-WT	This paper	N/A
IgG: 2554_01_D11-YTE	This paper	N/A
IgG: 2554_01_D11-WT	This paper	N/A
Fab: TPL0197_01_C08	This paper	N/A
Fab: TPL0544 _01_B01	This paper	N/A
Fab: TPL0039_05_B04	This paper	N/A
Fab: TPL0552_01_A05	This paper	N/A
Fab: 2555_01_A01	This paper	N/A
Fab: 2554_01_D11	This paper	N/A
anti-human IgG Fc	Sigma-Aldrich	I2136
Alkaline phosphatase-conjugated goat anti-human Fc	Sigma-Aldrich	A9544; RRID: AB_258459

**Bacterial and virus strains**		

TG1 Electrocompetent Cells	Lucigen	605022
*E. coli* strain BL21 (DE3)	New England Biolabs	C2527H

**Biological samples**		

Myotoxin II (Uniprot P24605)	Lomonte and Gutiérrez, 1989	N/A
α-cobratoxin (Uniprot P01391)	Latoxan	L8114

**Chemicals, peptides, and recombinant proteins**		

Platinum™ SuperFi II Green PCR Master Mix	Invitrogen	12369050
Ampicillin	Sigma Aldrich	A9518
EZ-Link™ NHS-PEG4-Biotin	Thermo Scientific	A39259
Skimmed milk powder	PanReac AppliChem (ITW Reagents)	A0830
Dynabeads™ M-280 Streptavidin	Invitrogen	11206D
Trypsin (phage display)	Sigma-Aldrich	T9201
Streptavidin	ThermoScientific	21135
TMB (3,3′,5,5′-Tetramethylbenzidine) substrate	Thermo Scientific	34021
Kinetic buffer	Sartorius	18–1105
Human FcRn biotinylated	Immunitrack	ITF01
Streptavidin-AP conjugate	Roche	11089161001
*p*-nitrophenyl-phosphate substrate	Merck	P4744
MCDB131 medium	Gibco	10372–019
L-glutamine solution	Sigma	G7513
Penicillin-Streptomycin	Sigma-Aldrich	P4458
Fetal Bovine Serum	Sigma-Aldrich	F7524
Mouse epidermal growth factor	Gibco	PMG8043
Hydrocortisone	Sigma-Aldrich	H0888
Geneticin	Gibco	10131–027
Blasticidin S HCl	Gibco	A1113903
Hank’s balanced salt solution	ThermoFisher	14025100
MEM non-essential amino acids 100X	Gibco	11140–035
RIPA buffer	ThermoFisher	89901
1x HBS-EP+ pH 7.4	Cytiva	BR100669
Amine coupling kit, type 2 (containing EDC, NHS, Ethanolamine)	Cytiva	BR100633
Dulbecco′s Phosphate Buffered Saline	Sigma-Aldrich	D8537

**Deposited data**		

Antibody sequences	This paper	https://data.mendeley.com/preview/j2rnfk6xj8?a=782d609d-674a-4139-8002-921b2a01170d
Raw data for all included figures and supplementary figures	This paper	https://data.mendeley.com/preview/j2rnfk6xj8?a=782d609d-674a-4139-8002-921b2a01170d

**Experimental models: Cell lines**		

HMEC-1 stably expressing HA-hFcRn-EGFP (HMEC-1-hFcRn)	Weflen et al.^22^	Harvard Medical School and Harvard Digestive Diseases Center

**Oligonucleotides**		

Primer: pSANG10 PelB FWD CGCTGCCCAGCCGGCCATGG	This paper	N/A
Primer: HLINK3 REV CTGAACCGCCTCCACCACTCGA	This paper	N/A

**Recombinant DNA**		

Plasmid containing scFv: pSANG10-3F-TPL0197_01_C08	Tulika et al.^38^	N/A
Plasmid containing scFv: pSANG10-3F-TPL0039_05_B04	Sørensen et al.^44^	N/A
Plasmid containing scFv: pSANG10-3F-TPL0039_05_B12	Sørensen et al.^44^	N/A
Plasmid: pIONTAS1	Schofield et al.^51^	N/A
Plasmid: pSANG10-3F	Martin et al.^70^	N/A

**Software and algorithms**		

ForteBio’s data analysis software version 12.2.2.4	ForteBio	https://www.sartorius.com/en/applications/life-science-research/label-free-detection/octet-support/software-download-request
GraphPad Prism9	GraphPad Software Inc	https://www.graphpad.com/scientific-software/prism/
ColabFold	ColabFold	https://github.com/sokrypton/ColabFold
AMBER Simulation Software	AmberMD	RRID: SCR_014230
GROMACS Simulation Software	GROMACS MD	http://www.gromacs.org/
PyMOL	Schrodinger, LLC	RRID: SCR_000305https://pymol.org
PR.Panta control (version 1.7.4)	NanoTemper Technologies GmbH	https://nanotempertech.com/
DiscoverMP (v2024 R1)	Refeyn Ltd.	https://www.refeyn.com/
Biacore™ Insight software (Control and Evaluation software)	Cytiva	29310602 https://www.cytivalifesciences.com/
iep calculator	Emboss	https://www.bioinformatics.nl/cgi-bin/emboss/iep?_pref_hide_optional=0

### Resource availability

#### Lead contact

Further information and requests for resources and reagents should be directed to and will be fulfilled by the lead contact, Andreas H. Laustsen (ahola@bio.dtu.dk).

#### Materials availability

All unique/stable reagents generated in this study are available from the lead contact upon reasonable request and a completed Materials Transfer Agreement.

#### Data and code availability

All raw data can be found in the Mendeley data repository. Accession codes are listed in the key resources table. Datasets are publicly available as of the date of publication. This paper does not report original code. Any additional information required to reanalyze the data reported in this paper is available from the lead contact upon reasonable request.

### Experimental model and study participant details

#### Microbe strains

TG1 Electrocompetent Cells (Lucigen) and *E. coli* strain BL21 DE3 (New England Biolabs).

#### Cell lines

HMEC-1 cells stably expressing HA-hFcRn-EGFP (HMEC-1-hFcRn).

### Method details

#### Toxins included in the study

Myotoxin II (M-II; Uniprot P24605), which has a molecular mass of 13,750 Da, was purified from the venom of *B. asper* by cation-exchange chromatography on a CM-Sephadex C25 column (20 × 2 cm), followed by reverse-phase high-performance liquid chromatography (RP-HPLC) using a C8 column (250 × 10 mm) as described previously.^49^ Briefly, the venom was fractioned on CM-Sephadex C25 column equilibrated with 0.05 M Tris, 0.1 M KCl, pH 7.0 buffer, at 0.4 mL/min and eluted using a gradient toward 0.75 M KCl in the same buffer. The last eluting peak was collected and after desalting further separated using RP-HPLC, where the elution was carried out at 2.5 mL/min with a gradient from water to acetonitrile, both containing 0.1% trifluoroacetic acid. Fractions of interest were collected, dried in a vacuum centrifuge at 45°C, and stored at −20°C α-cobratoxin (α-cbtx; Uniprot P01391) from *N. kaouthia*, with a molecular weight of 7,831 Da, was purchased (Latoxan) and reconstituted according to the manufacturer’s instructions.

#### Biotinylation of toxin

Purified M-II and α-cbtx were dissolved in phosphate buffered saline (PBS, pH 7.2) and biotinylated by adding EZ-Link NHS-PEG_4_-Biotin reagent (Thermo Scientific, A39259) to the toxin at 1:1.25 toxin:biotinylation reagent molar ratio and the mixture was incubated at room temperature for 30 min. The biotinylated toxins were separated from the free biotin on buffer exchange columns (Vivacon 500, Sartorius, 3000 Da Molecular Weight Cut-Off) according to the manufacturer’s protocol. Protein concentration was determined by measuring the absorbance at 280 nm with a NanoDrop One instrument (Thermo Scientific) and the concentration was calculated using the toxin’s extinction coefficients.

#### Engineering of pH-dependent antigen binding properties by light-chain shuffling

To engineer the pH-dependent antigen binding properties of single-chain variable fragments (scFvs) TPL0197_01_C08, TPL0039_05_B04, and TPL0039_05_B12, light-chain shuffling was carried out as described previously.^71^ Briefly, the variable heavy (V_H_) regions of scFvs TPL0197_01_C08, TPL0039_05_B04, and TPL0039_05_B12 were PCR amplified from the pSANG10-3F plasmid with pSANG10 PelB FWD (CGCTGCCCAGCCGGCCATGG) and HLINK3 REV (CTGAACCGCCTCCACCACTCGA) primers using Platinum SuperFi II Green PCR Master Mix (Invitrogen), digested, purified, and ligated into pIONTAS1^51^ vectors containing naive variable light (V_L_) lambda and kappa chain libraries. Electrocompetent TG1 cells (Lucigen) were transformed by electroporation (BioRad MicroPulser) and plated on 2TY agar plates supplemented with 2% glucose and 100 μg/mL ampicillin following phenotypic expression. Dilutions of transformed cells were plated to determine the library sizes, and the correct heavy chain insert was determined by colony PCR.

#### Library rescue and solution-based phage display selections

Phage rescue from the chain shuffled libraries, deselection of streptavidin-binding phages, and three rounds of phage display selections were performed as described previously^71^ with the following modifications: to enrich for antibodies with pH-dependent antigen binding properties, all three rounds of selections included a deselection of phages that bound the antigen at pH 5.5. To do this, phages were incubated with biotinylated antigen for 30 min in 3% (w/v) milk PBS pH 5.5, followed by the addition of Dynabeads (Invitrogen, M-280) for 15 min to capture the biotinylated antigen-bound phage complexes that were formed at pH 5.5. The mixture was then placed on a magnetic rack to separate the captured antigen-phage complexes, while the solution containing unbound phages (that did not bind the antigen at low pH) was collected. The pH of this unbound phage-containing solution (3% (w/v)milk PBS) was adjusted to pH 7.4 using 1 M Tris (pH 8.0). These phages, now in a neutral pH solution, were employed for selection by allowing binding to biotinylated antigen,^71^ followed by elution of the bound phages using PBS at pH 5.5 for 15 min. The eluted phages were trypsinated to reduce the population of bald phages capable of infecting the bacterial cells.^72^ The concentrations of α-cbtx and M-II during the deselection step (antigen binding at pH 5.5) and selection step (antigen binding at pH 7.4) in all three rounds were 10 nM and 1 nM, respectively.

#### ELISA assessment of polyclonal phage outputs

The phage outputs were evaluated for antigen binding in enzyme-linked immunosorbent assays (ELISAs). Selected phage pools were left to bind biotinylated toxins (10 μg/mL) captured on streptavidin-coated MaxiSorp plates (Thermo Scientific). After washing with PBS supplemented with 0.1% (v/v) Tween 20 (PBS-T 0.1%) and PBS, bound phages were detected using a 1:2000 dilution of anti-M13 horseradish peroxidase (HRP) antibody (Sino Biological) and 3,3′,5,5′-Tetramethylbenzidine (TMB) (Thermo Scientific) according to the manufacturer’s protocol.

#### Sub-cloning, screening of scFvs, and sequencing

Sub-cloning of scFv encoding genes from phage outputs from the third round of selections into the pSANG10-3F expression vector was performed as described previously.^73^ From each of the selection outputs, 184 colonies were picked, expressed in 96 well format, and assessed for binding to 10 nM of their respective toxins in an expression-normalized capture dissociation-enhanced lanthanide fluorescence immunoassay (ENC DELFIA) as described previously.^73^ Clones showing a binding signal 10 times above the background (10,000 TRF) were designated as binding clones.

#### Reformatting and production of Fab and full-length IgG1 variants

The reformatting of the scFvs to Fab and IgG1 variants and their production was performed as described previously,^38^ except that the expression vectors containing human lambda light chain were used and that some IgG1s were produced both with a wild-type Fc region and encompassing LALA^74^ and YTE^15^ amino acid substitutions (L234A/L235A and M252Y/S254T/T256E, respectively). The LALA mutation is known to decrease the binding to Fc gamma receptors, which was, however, not evaluated in this study. In this text, IgG1-YTE refers to IgGs with both substitutions.

#### Biolayer interferometry (BLI) off-rate screening of Fabs in crude expression media

BLI experiments were performed on an OctetRed 96 system (ForteBio). Streptavidin (SAX) biosensors (Sartorius) were blocked for at least 10 min in 1 × Kinetics Buffer (KB) (PBS-T 0.05% and 0.1% BSA, Forte Bio). Screening was performed by loading biotinylated α-cbtx or M-II toxin (1 μg/mL, pH 7.4) on SAX biosensors. The loaded biosensors were transferred into Fab-containing expression media for 300 s, and dissociation was performed for 300 s in 1× HEPES-MES buffer at either pH 7.4 or pH 5.5. The biosensors were regenerated at the end of each cycle by iteratively dipping them into 10 mM Glycine pH 2.0 and 1 × KB (5 cycles, of 10 s). The experiment was performed at 25°C with shaking at 1000 rpm. A control with no antibody addition was included and used for baseline subtraction. ForteBio’s data analysis software (version 12.2.2.4) was employed to obtain dissociation rates for pH 7.4 and 5.5 using a 1:1 binding model with local fitting.

#### BLI off-rate screening of purified Fabs and IgG1s over a range of pH values

The off-rates of the purified Fabs to their cognate toxins over a range of pH values were obtained as described above, with a few modifications. Briefly, 700 nM of Fab was prepared in HEPES-MES buffer at pH 7.4, and the association and dissociation steps were carried out for 180 s and 600 s, respectively. A total of 8 cycles were performed, in which the association conditions were kept the same, while the pH of the dissociation buffer (HEPES-MES) changed to: 7.4, 6.5, 6.0, 5.5, 5.0, 4.5, 4.0, and 3.5 respectively. The tips were regenerated in 10 mM Glycine pH 2.0 for 10 s x 7 cycles in between the rounds. ForteBio’s data analysis software (version 12.2.2.4) was employed to obtain dissociation rates at the different pH values (using a 1:1 binding model with local fitting). The off-rates of IgG1s at pH 7.4 and 5.5 were determined as described above except that 10 nM of IgG1 was used for association.

#### BLI determination of kinetic constants of Fabs

The binding constants, K_D_, of the purified Fabs at pH 7.4 and 5.5 were determined as described above, except that the Fabs were used for association in a 2-fold- dilution series ranging from 3 to 250 nM. Association was performed in HEPES-MES buffer pH 7.4 for 300 s and dissociation was performed in HEPES-MES buffer pH 7.4 and 5.5 for 600 s. ForteBio’s data analysis software was used to derive the kinetic constants (using a 1:1 binding model with global fitting).

#### Surface plasmon resonance (SPR) determination of kinetic constants of IgG1s

The binding constants, K_D_, of the purified IgGs at pH 7.4 and 5.5 were determined using SPR (Biacore 8K, Cytiva). The IgG1s were immobilized onto the CM5 (Series S, Cytiva) surface using a standard amine coupling procedure at 25°C. Each IgG was diluted to 20 μg/mL in the immobilization buffer (10 mM sodium acetate, pH 4). The four channels, each containing a reference and measurement flow cell (flow cell 1 and 2) clamped against the CM dextran chip surface were simultaneously activated by injecting a 1:1 mixture (*v/v*) of EDC/NHS at a flow rate of 10 μL/min. Across all channels, flow cell 1 was left blank as a reference surface to monitor non-specific binding and each channel had a different IgG1 immobilized in flow cell 2. Each IgG1 diluted in immobilization buffer was injected in an independent channel of the flow cell 2. After a sufficiently high level of IgG (i.e., >1000 RU) was reached (Table S5) the immobilization was terminated and the remaining active sites on the chip surface were blocked by injecting 1M ethanolamine preventing electrostatic interactions with the CM dextran surface.

To determine the pH dependency of the IgG1s, α-cbtx diluted in HBS-EP (pH 7.4) in the concentration range of 0.2–200 nM was injected across channels 1–4 at a flow rate of 30 μL/min and allowed to associate to the IgGs for 120 s. After association, running buffer with a pH of 7.4 or 5.5 was injected at a flow rate of 30 μL/min for 300 s to determine whether the antigen was released by the IgG in these different pHs. Between each cycle, the surface was regenerated by injected 30 μL of 10 mM glycine-HCl (pH 2) for 30 s.

A kinetic titration assay was run to determine the kinetic parameters of the anti α-cbtx antibodies toward α-cbtx in two different pHs. First, the running buffer (either HBS-EP pH 7.4, or HBS-EP pH 5.5) was injected at a flow rate of 30 μL/min across channels 1–4 in flow cells 1–2 to serve as a blank. The buffer had a contact time of 120 s and a dissociation time of 60 s. Following this, α-cbtx diluted in the concentration range of 0.2–200 nM in the running buffer with pH 7.4 or 5.5 was sequentially injected in increasing concentration across channels 1–4 in flow cells 1–2. Each antigen concentration had a contact time of 120 s, with no regeneration in between. The antigen was allowed to dissociate in the system running buffer (pH 7.4 or 5.5) over a prolonged time of 7000 s for pH 7.4 or 4000 s for pH 5.5. Using the Biacore insight evaluation software the data was fitted using a 1:1 binding model and a 1:1 dissociation model, with local fitting to obtain kinetics data (Table S4).

#### Biophysical characterization of IgG1s and antibody-antigen complexes

The thermal stability of the IgGs was assessed by determining their melting temperature (T_M_) using differential scanning fluorimetry on a Prometheus Panta instrument (NanoTemper Technologies GmbH). Antibodies at a concentration of 25 mg/mL in PBS pH 7.4 were loaded in 10 μL glass capillaries (standard grade capillaries from NanoTemper Technologies GmbH). The samples were subjected to a temperature ramp from 25°C to 110°C using a slope of 2°C/min. The fluorescence emission at 330 and 350 nm was measured. Melting temperatures were determined using the PR.Panta Control software (version 1.7.4, NanoTemper Technologies GmbH).

The sizes of the IgG-antigen complexes were evaluated using mass photometry on a Refeyn TwoMP instrument (Refeyn Ltd.). Antibodies and antigens were diluted in PBS pH 7.4 and pH 6 mixed to a final concentration of 33 nM for the IgG and 667 nM for the antigen, and then incubated for an hour at room temperature before measurement. Measurements were done in 6 well sample cassettes placed on MassGlass UC cover slips (both purchased from Refeyn Ltd.). Samples were measured using the buffer-free focus mode of the instrument, by adding 15μL of sample to each well. Videos were recorded for 60 s at a rate of 48 frames per second (fps). Landing events were detected using the Discover MP software (version 2024 R1, Refeyn Ltd.). Molecular masses were calibrated using standard samples of 20 nM BSA (66.5 kDa) and 20 nM IgG (150 kDa). To evaluate the mass of the dominant species in each acquisition, the probability density of the detected events was estimated using the stats.Gaussian_kde function of scipy (version 1.10.1). The point of highest density was then found using the signal.find_peaks function of scipy (version 1.10.1).

#### Molecular dynamics simulations to structurally characterize the influence of changes in protonation

Artificial intelligence-based tools, such as AlphaFold2 (AF2) and RoseTTAFold, have revolutionized the field of protein structure prediction.^75^^,^^76^ ColabFold has even further increased the accessibility of these protein structure prediction tools by combining AF2 with the fast homology search MMseqs2, making it an easy to use and fast software (∼90-fold speed up in prediction) to predict homo-and heteromeric complexes, matching the prediction quality of AF2 and AF-multimer.^77^

Here, we used ColabFold in combination with classical molecular dynamics (MD) simulations to predict and characterize the protein-protein interface of the parent and light-chain shuffled antibodies binding to α-cbtx or M-II respectively. To identify the most probable protonation states of the antibodies with and without the antigen present at pH 7.5 and pH 5.0, we performed each 3 × 100 ns of constant pH simulations using the implementation for explicit solvent in the AMBER by Roitberg and coworkers.^78^ In this constant pH approach, the simulation is interrupted at periodic intervals and protonation changes are attempted based on a Monte Carlo Metropolis criterion. To neutralize the charges, the uniform background charge was used, which is required to compute long-range electrostatic interactions.^79^ Using the tleap tool of the AmberTools22^80^ package, the structures were soaked in cubic water boxes of TIP3P water molecules with a minimum wall distance of 12 Å to the protein.^81^^,^^82^^,^^83^^,^^84^ For all simulations, parameters of the AMBER force field 14SB were used.

For the complexes C08, B01, B04, and A05 with the respective toxin at pH 7.5, pH 5.5, and pH 5.0, three repetitions of 1 μs of classical molecular dynamics simulations were performed. Molecular dynamics simulations were performed in an NpT ensemble using pmemd.cuda.^85^ Bonds involving hydrogen atoms were restrained by applying the SHAKE algorithm^86^ allowing a time step of 2 fs. Atmospheric pressure of the system was preserved by weak coupling to an external bath using the Berendsen algorithm.^87^ The Langevin thermostat was used to maintain the temperature during simulations at 300 K. The interaction energies were calculated with cpptraj using the linear interaction energy (LIE) tool. The electrostatic and van der Waals interaction energies were calculated for all frames of each simulation and provided the simulation-averages of these interactions. PyMOL was used for visualizing protein structures (The PyMOL Molecular Graphics System, Version 2.5.2 Schrödinger, LLC).

#### IgG1 binding to hFcRn in ELISA

ELISA for determination of pH-dependent binding of IgG1s to human FcRn (hFcRn) was performed as described previously.^88^ Briefly, ELISA plates (Costar) were coated with different amounts of IgG1s (0.1–6.7 nM) in PBS and incubated overnight at 4°C. Biotinylated, truncated, monomeric hFcRn (hFcRn-bio) (Immunitrack) was incubated with alkaline phosphatase (AP)-conjugated streptavidin (Roche), added to the plates at final concentrations of 0.25 μg/mL hFcRn-bio and 3.36 μg/mL of streptavidin-AP before the binding was detected using a *p*-nitrophenyl-phosphate substrate (Sigma-Aldrich) and absorbance at 405 nm was measured in a Sunrise spectrophotometer (Tecan).

#### IgG1 binding to hFcRn in analytical affinity chromatography

Analytical hFcRn affinity chromatography was performed to measure receptor binding of the IgG1s throughout a pH gradient using an ÄKTA Avant25 instrument (GE Healthcare), as described previously.^53^^,^^54^ Briefly, 77 μL of a 1 mg/mL IgG1 solution was injected in a pH 5.5 buffer (20 mM MES, 140 mM NaCl; Sigma-Aldrich), and eluted by a linear gradient to pH 8.8 (20 mM Tris-HCl, 140 mM NaCl; Sigma-Aldrich) over 110 min. To study IgG1-antigen complexes, cognate antigen (M-II or α-cbtx, respectively) and IgG1s (1 mg/mL) were preincubated for 20 min at room temperature at a 2:1 M ratio in buffer A (20 mM MES, 140 mM NaCl) to ensure that binding equilibrium had occurred, before dilution into a total volume of 85 μL buffer A (pH 5.5) and column application. To determine the elution pH at a particular retention time, the pH was monitored by a pH detector (GE Healthcare).

#### Analytical size-exclusion chromatography

Analytical SEC was performed using a Superdex 200 increase 10/300 GL column (Cytiva) coupled to an ÄKTA avant 25 (Cytiva). Injections were performed in 1× PBS (Gibco) and 10 μg of the individual antibodies was injected in a total volume of 10 μL.

#### Sequence based net protein charge calculations

Sequence based net charge of the antibodies at pH values between 4 and 10, were calculated using the Emboss iep calculator (http://www.bioinformatics.nl/cgi-bin/emboss/iep?_pref_hide_optional=0). All cysteines were assumed to form disulfide bridges. Whole Fvs were defined as the combined variable heavy chain (HC) and variable light chain (LC) sequences. Remaining residues were defined as the framework after removing CDRs, and were assumed to have only one N-terminal residue. CDRs were not assumed to have any terminal residues.

#### Human endothelial cell-based recycling assay (HERA)

HERA experiments were performed to quantify the amount of IgG1 and IgG1-antigen complexes taken up, recycled, and retained in the cells, as described previously.^53^ HMEC-1 cells stably expressing hFcRn N-terminal tagged with hemagglutinin and C-terminal tagged with enhanced green fluorescent protein (HA-FcRn-EGFP), HMEC-1-FcRn cells,^22^ were used for the experiments. The cells were cultured at 37°C and 8% CO_2_ in MCDB131 medium (Gibco) supplemented with 2 mM L-glutamine (Sigma), 25 μg/mL streptomycin, 25 U/mL penicillin (Sigma-Aldrich), 10% FCS (Sigma-Aldrich), 10 ng/mL mouse epidermal growth factor (Gibco), 1 μg/mL hydrocortisone (Sigma-Aldrich), and 100 μg/mL G418 (Gibco) and 50 μg/mL blasticidin (Gibco) to maintain receptor expression.

1.5·10^5^ HMEC-1-FcRn cells were seeded in 250 μL of culturing medium per well in two 48-well plates (Costar) (Uptake and Residual plate). 20–24 h after seeding, the medium was removed, and the cells were washed twice in 300 μL of pre-warmed Hank’s balanced salt solution (HBSS; ThermoFisher). Cells were starved at 37°C for 1 h in pre-warmed HBSS before 800 nM of the IgG1 variants or IgG1-biotinylated antigen complex at 1:2 molar ratio (incubated at RT for 20 min) were diluted in pre-warmed HBSS and added to the cells at a final volume of 125 μL/well in triplicates. After a 3-h incubation, the samples were removed, and the cells were washed four times in 250 μL ice-cold HBSS to remove extracellular IgG1 or IgG1-antigen complexes. Uptake plates were frozen at −80°C following aspiration of washing medium, while 220 μL/well of pre-warmed serum-free growth medium supplemented with 1× MEM non-essential amino acids (Gibco) were added to the recycling plates. After another 3-h incubation, recycling samples were harvested and frozen at −20°C. Residual plates were washed four times with ice-cold HBSS and frozen at −80°C. On the day of analysis, frozen cells were lysed by adding 220 μL/well of RIPA buffer (ThermoFisher) supplemented with 1× complete protease inhibitor cocktail (Roche) and incubated on a shaker for 10 min on ice. Cellular debris was removed by 5 min centrifugation at 10,000 × g and the amount of IgG1 and IgG1-antigen complexes was determined with ELISA.

#### Quantification of IgG1 in uptake, residual, and recycling samples in ELISA

Detection of IgG1 present in the lysates (uptake and residual samples) and recycling media was quantified by a two-way anti-Fc ELISA. 96-well plates (Costar) were coated with anti-human IgG Fc (Sigma) diluted 1:1000 in PBS and incubated overnight at 4°C. The next day, plates were blocked with 250 μL/well of milk prepared in PBS-T 0.05% and washed four times with PBS-T 0.05%. Cell lysates and medium samples were added to the plates and to quantify the protein amounts, standards with 2-fold serial dilutions (0.342–350 ng/mL) of the IgG1s were included. Following 1.5-h incubation at room temperature, an AP-conjugated goat anti-human Fc antibody (Sigma-Aldrich), diluted 1:5000 in milk PBS-T 0.05% was added and incubated for 1 h before substrate addition and reading as described above.

#### Quantification of IgG1-antigen complexes in uptake, residual, and recycling samples in ELISA

Detection of the antigen-antibody complexes was performed as described for IgG1s except that plates were coated with 10 μg/mL of streptavidin diluted in PBS overnight at 4°C instead of anti-human IgG.

Two or three independent HERA experiments were performed with three replicates of each sample, and numerical data were summarized as the mean ± SD using GraphPad Prism9 software (San Diego, CA). For the IgG1-antigen complex experiments, values were normalized relative to the uptake values of each protein. Each global mean was compared using an unpaired Student’s t test. Two-tailed *p*-values ≤0.05 were considered statistically significant.

### Quantification and statistical analysis

GraphPad Prism 9 Software (GraphPad Software, Inc.) was used to analyze raw data and for statistical analysis (unpaired student’s t-tests) with a 95% confidence level, and *p* < 0.05 was defined as statistically significant. Figures were prepared by the same program or created with BioRender.

## Supplemental information

Document S1. Figures S1–S8 and Tables S1–S5

Document S2. Article plus supplemental information
