# Supplementary material for: Engineering of pH-dependent antigen binding properties for toxin-targeting IgG1 antibodies using light-chain shuffling
Source: Structure. 2024 Sep 5;32(9):1404–1418.e7. doi: 10.1016/j.str.2024.07.014 (PMC11385703; doi:10.1016/j.str.2024.07.014)
Supplement: Document S1. Figures S1–S8 and Tables S1–S5 [file mmc1.pdf]

Structure, Volume *32*

Supplemental Information

Engineering of pH-dependent antigen binding properties for toxin-targeting IgG1 antibodies using light-chain shuffling

Tulika Tulika, Fulgencio Ruso-Julve, Shirin Ahmadi, Anne Ljungars, Esperanza Rivera-de-Torre, Jack Wade, Monica L. Fernández-Quintero, Timothy P. Jenkins, Selma B. Belfakir, Georgina M.S. Ross, Lars Boyens-Thiele, Alexander K. Buell, Siri A. Sakya, Christoffer V. Sørensen, Markus-Frederik Bohn, Line Ledsgaard, Bjørn G. Voldborg, Chiara Francavilla, Tilman Schlothauer, Bruno Lomonte, Jan Terje Andersen, and Andreas H. Laustsen

Supplementary materials for

Engineering of pH-dependent antigen binding properties for toxin-targeting IgG1 antibodies using light-chain shuffling

Tulika Tulika[1], Fulgencio Ruso-Julve[2,3,4], Shirin Ahmadi[1], Anne Ljungars[1], Esperanza Rivera-de-Torre[1], Jack Wade[1], Monica L. Fernández-Quintero[1], Timothy P. Jenkins[1], Selma B. Belfakir[1,5], Georgina M.S. Ross[5], Lars Boyens-Thiele[1], Alexander K. Buell[1], Siri A. Sakya[2,3,4], Christoffer V. Sørensen[1], Markus-Frederik Bohn[1], Line Ledsgaard[1], Bjørn G. Voldborg[1], Chiara Francavilla[1], Tilman Schlothauer[6], Bruno Lomonte[7], Jan Terje Andersen[2,3,4]*, Andreas H. Laustsen[1]*

[1]Department of Biotechnology and Biomedicine, Technical University of Denmark, Lyngby, Denmark
[2]Department of Pharmacology, University of Oslo, Oslo, Norway
[3]Department of Immunology, Oslo University Hospital Rikshospitalet, Oslo, Norway
[4]Precision Immunotherapy Alliance (PRIMA), University of Oslo, Oslo, Norway
[5]VenomAid Diagnostics ApS, Lyngby, Denmark
[6]Roche Pharma Research and Early Development (pRED), Roche Innovation Center Munich, Penzberg, Germany
[7]Instituto Clodomiro Picado, Facultad de Microbiologia, Universidad de Costa Rica, San Jose, Costa Rica

*Correspondence:
Andreas H. Laustsen, ahola@bio.dtu.dk; Jan Terje Andersen, j.t.andersen@medisin.uio.no
Lead contact: Andreas H. Laustsen, ahola@bio.dtu.dk

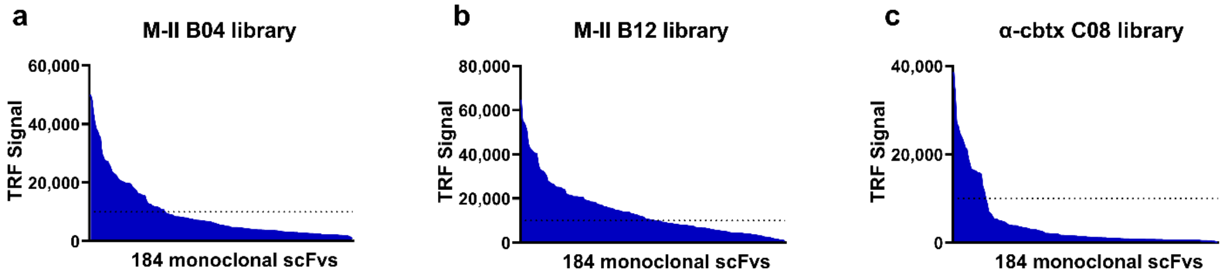

Figure S1. Primary screening of monoclonal scFvs obtained from phage display selection campaigns, related to Figure 1. Binding signal of 184 monoclonal scFvs from the library **(a)** B04, **(b)** B12, and **(c)** C08 against their target antigen. Clones showing a binding signal above the set threshold of 10,000 units (shown by dotted lines) were selected for further analysis.

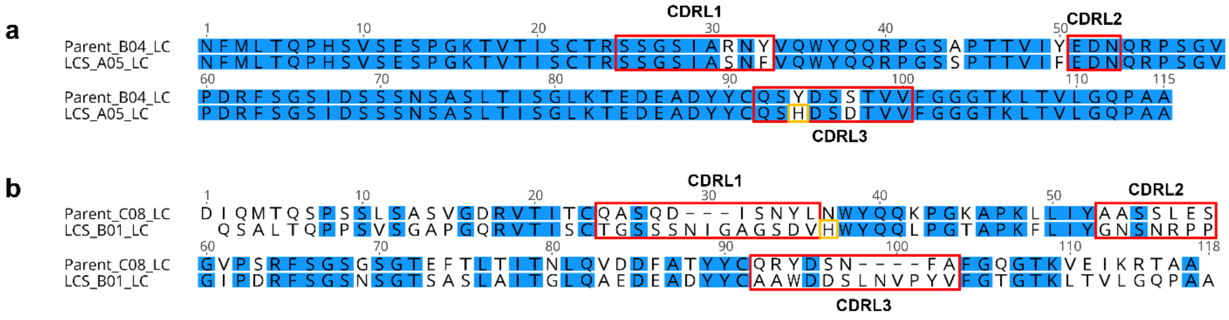

Figure S2. Light chain sequence alignments of parental and light-chain shuffled antibodies, related to Figure 2. (a) Sequence alignment of light chains of the anti-M-II parent antibody B04 and the light-chain shuffled antibody A05. **(b)** Sequence alignment of light chains of the anti-α-cbtx parent antibody C08 and the light-chain shuffled antibody B01. Amino acid residues that are identical in the sequence alignments are marked with a blue background, while distinct amino acids are marked with a white background. The light-chain CDRs are shown in red boxes and the gained histidine residues in the light-chain shuffled antibodies are shown in yellow boxes. The sequence alignments were made using Geneious Prime. (LCS = Light-chain shuffled, LC = Light chain, CDRL= light chain complementarity-determining region).

a

Electrostatic interaction energies / kcal/mol				
**Antigen**	**Antibody**	**pH 7.5**	**pH 5.5**	**pH 5.0**
α-cbtx	C08	-282 ± 28	-151 ± 37	-151 ± 37
α-cbtx	B01	-189 ± 52	-125 ± 53	-85 ± 29
M-II	B04	-453 ± 53	-392 ± 42	-307 ± 42
M-II	A05	-470 ± 51	-268 ± 68	-173 ± 24

b

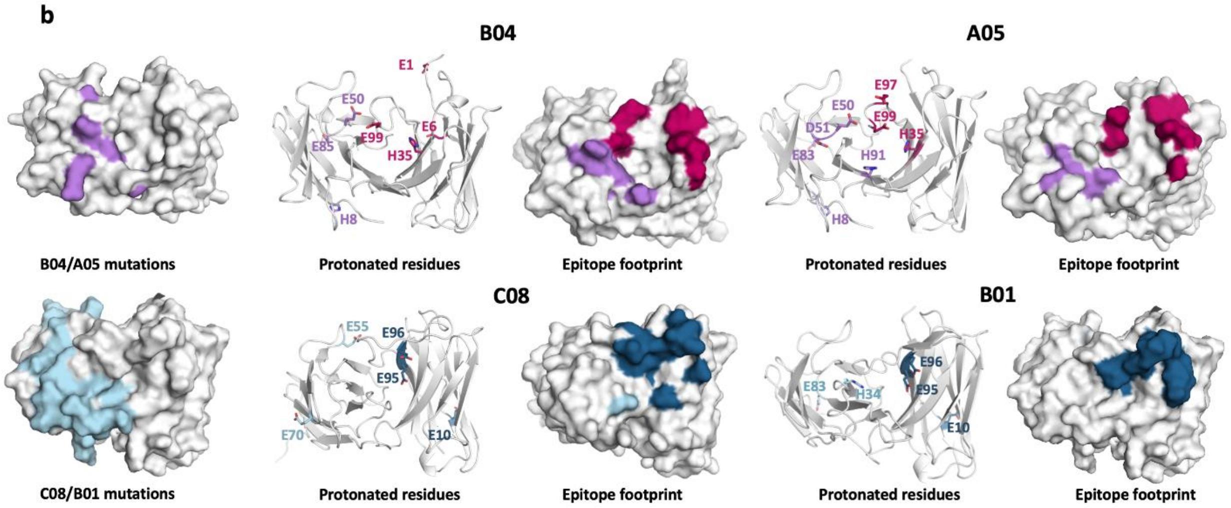

Figure S3: Structural characterization of the influence of changes in protonation at pH 5.5 and pH 7.4 on antigen binding, related to STAR methods.

(a) Electrostatic interaction energies at pH 7.5, pH 5.5, and pH 5.0 for the antibodies C08 and B01 binding to α-cbtx and B04 and A05 binding to M-II. **(b)** The differences in the light chain between the variants are depicted in light purple for B01/A05 and in light blue for B04/A05 on the surface of the structures. For each antibody, the protonated residues at pH 5.5 are depicted and highlighted as sticks and color-coded based on their location in the heavy and light chains respectively. Additionally, the predicted antigen-binding sites (epitope fingerprints) are color-coded in shades of blue for α-cbtx and in shades of purple for M-II.

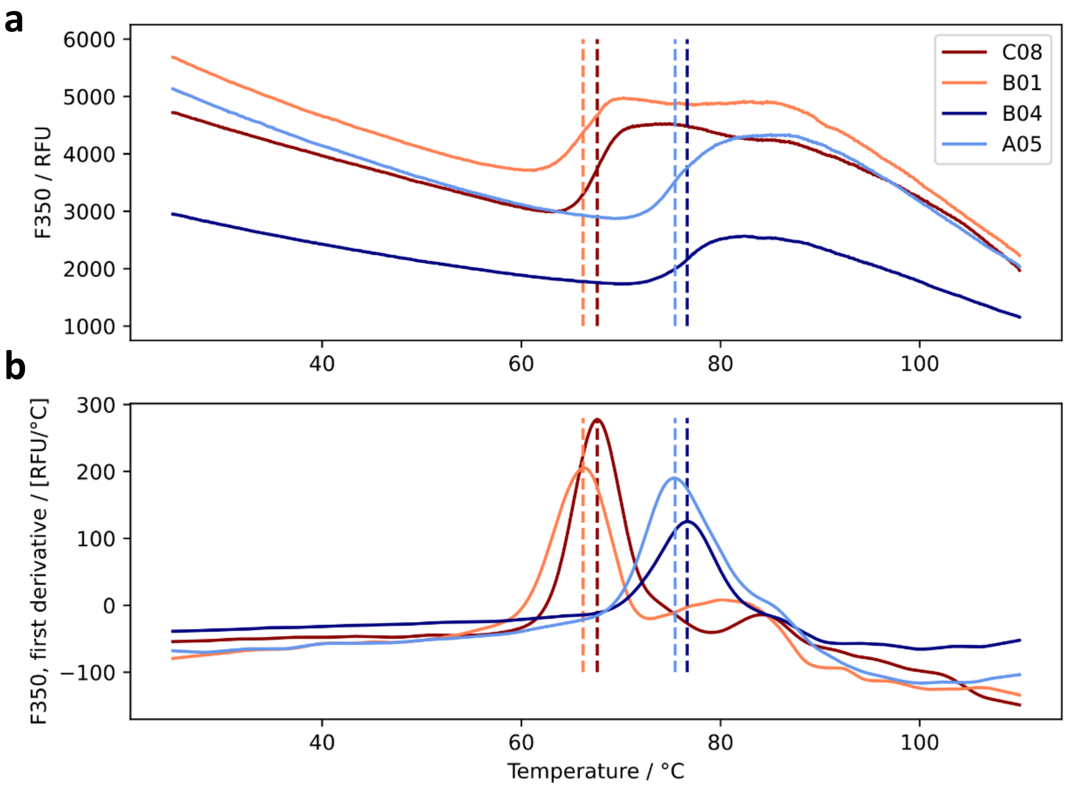

Figure S4. Differential scanning fluorimetry of parental (C08 and B04) and light-chain shuffled (B01 and A05) antibodies, related to STAR methods. (a) Fluorescence emission at 350 nM plotted against temperature. **(b)** The slope of the fluorescence emission at 350 nM plotted against temperature. **(a-b)** Dashed vertical lines indicate the detected melting temperatures (T_M).

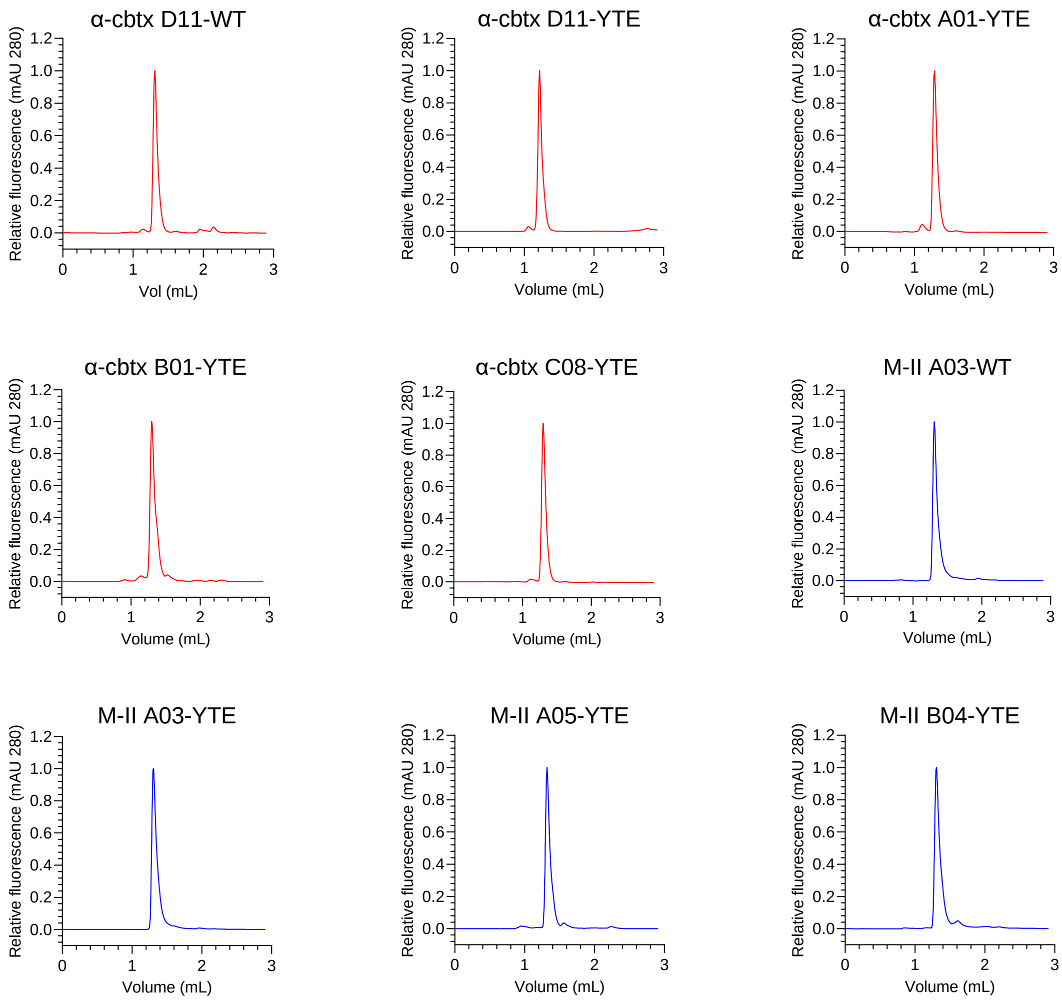

Figure S5. Analytical size exclusion chromatogram elution profiles of IgGs, related to STAR methods.

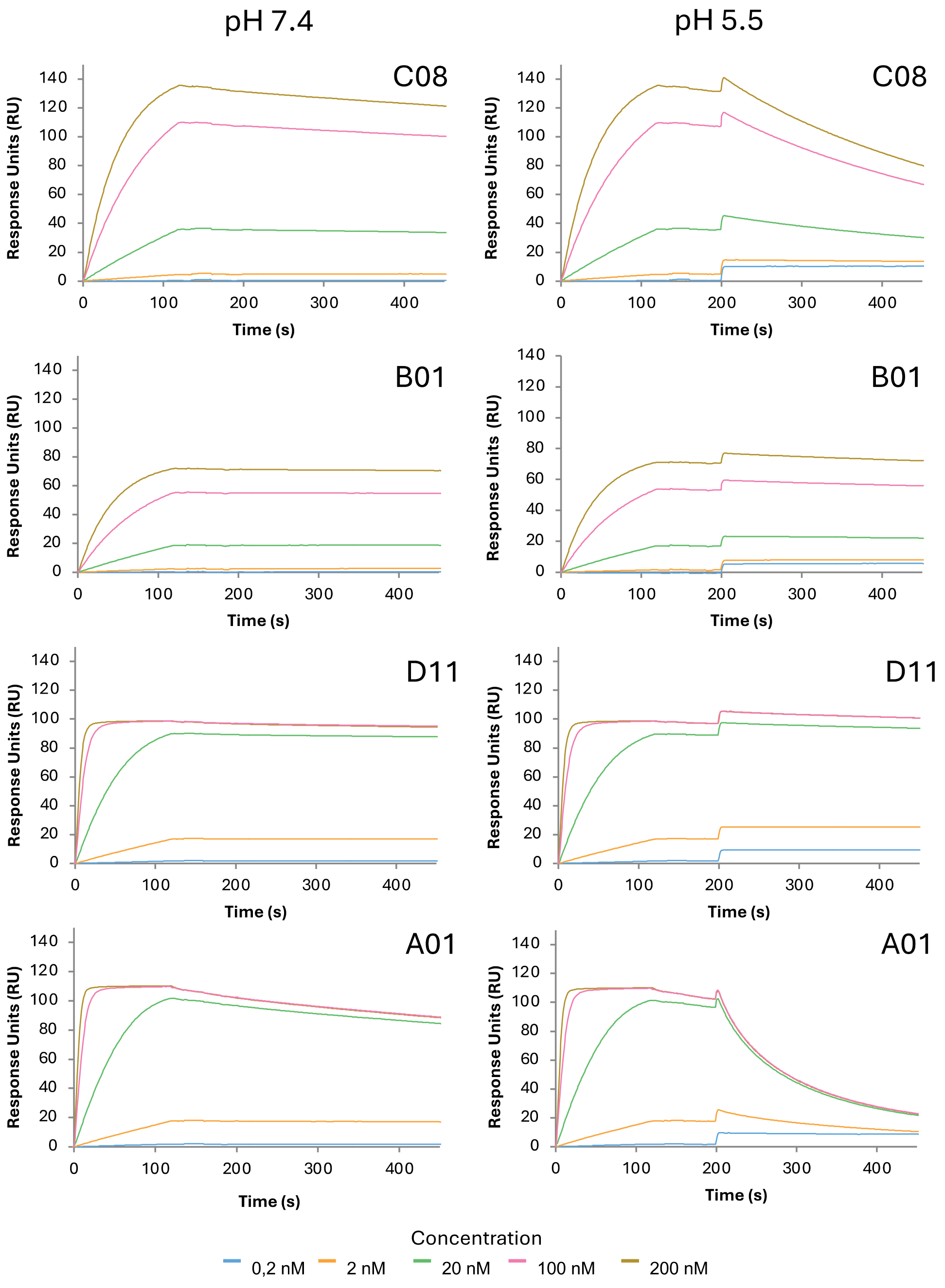

Figure S6: Association and dissociation of α-cbtx to IgGs at pH 7.4 and pH 5.5 measured using surface plasmon resonance (SPR), related to Figure 3. Sensorgrams showing the association of α-cbtx (0.2 – 200 nM) to IgGs at pH 7.4 and dissociation at pH 7.4 and pH 5.5. A slight jump in RU can be observed at 200 seconds, which is due to a shift in the refractive index

when the running buffer is switched from pH 7.4 to 5.5 after the end of the antigen injection phase (at 120s).

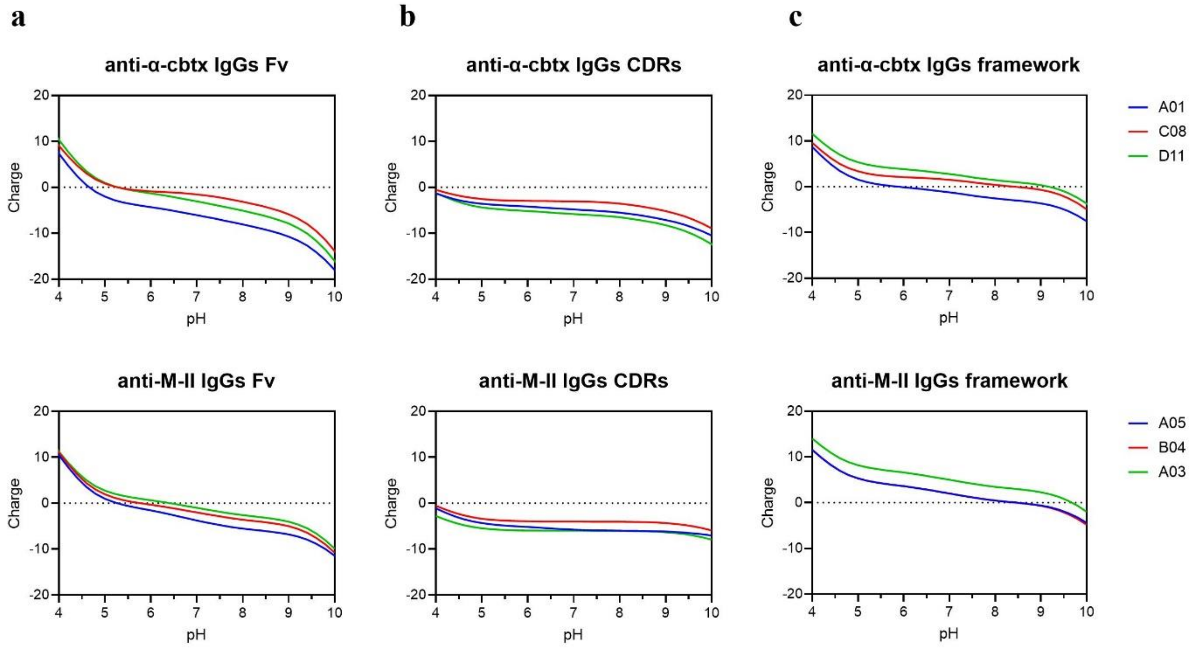

Figure S7. Net charge of the variable regions of the anti-α-cbtx and anti-M-II antibodies, related to Figure 3. The charge of **(a)** whole variable regions (Fvs), **(b)** complementary-determining regions (CDRs), and **(c)** frameworks across a pH gradient was calculated by using EMBOSS iep software.

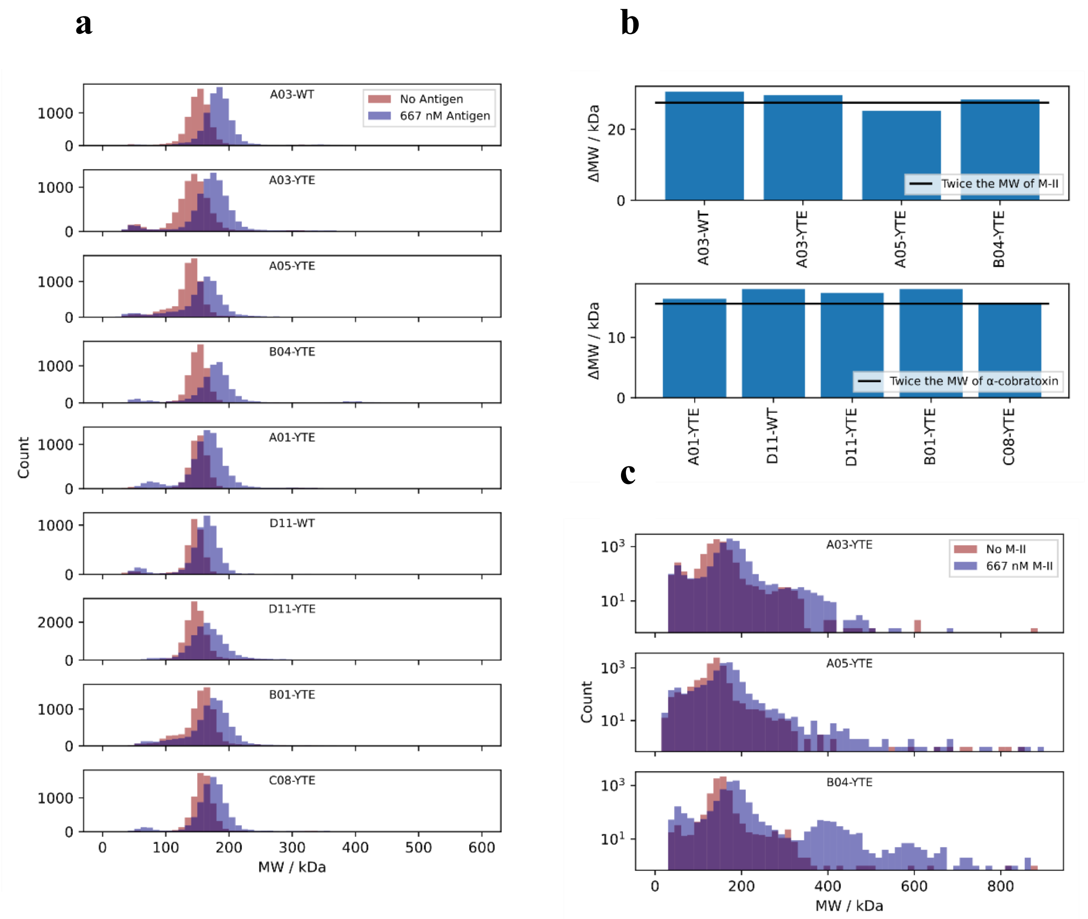

Figure S8: Mass photometry of antibody-antigen complexes, related to Figure 3. (a) Histograms of detected masses of antibodies at 33 nM, with (blue) and without (red) the presence of 667 nM of either M-II or α-cbtx. **(b)** Bar charts of the observed difference in mass of the dominant species upon addition of antigen. Black horizontal lines indicate the mass of two antigens. **(c)** Log-scaled histograms of detected masses of three anti-M-II antibodies at 33 nM with (blue) and without (red) 667 nM M-II.

Table S1: Off-rates of Fabs at pH 7.4 and pH 6.5, 6.0, 5.5, 5.0, 4.5, 4.0, and 3.5 as determined by BLI, related to Figure 2.

**pH**	**α-cbtx targeting Fabs k_{off} (s^{-1})**				**M-II targeting Fabs k_{off} (s^{-1})**		
	**A01 (PC)**	**C08 (Parent)**	**B01 (LCS)**	**D11 (NC)**	**B04 (Parent)**	**A05 (LCS)**	**A03 (NC)**
**7.4**	$9.5 \cdot 10^{-04}$	$6.9 \cdot 10^{-04}$	$8.1 \cdot 10^{-04}$	$2.6 \cdot 10^{-04}$	$7.1 \cdot 10^{-03}$	$9.7 \cdot 10^{-03}$	$6.9 \cdot 10^{-04}$
**6.5**	$1.8 \cdot 10^{-03}$	$7.7 \cdot 10^{-04}$	$7.1 \cdot 10^{-04}$	$2.7 \cdot 10^{-04}$	$7.5 \cdot 10^{-03}$	$1.3 \cdot 10^{-02}$	$5.8 \cdot 10^{-04}$
**6.0**	$3.3 \cdot 10^{-03}$	$1.5 \cdot 10^{-03}$	$7.0 \cdot 10^{-04}$	$3.0 \cdot 10^{-04}$	$8.5 \cdot 10^{-03}$	$1.8 \cdot 10^{-02}$	$6.9 \cdot 10^{-04}$
**5.5**	$1.2 \cdot 10^{-03}$	$4.1 \cdot 10^{-03}$	$7.7 \cdot 10^{-04}$	$3.9 \cdot 10^{-04}$	$1.4 \cdot 10^{-02}$	$3.9 \cdot 10^{-02}$	$1.2 \cdot 10^{-03}$
**5.0**	$1.9 \cdot 10^{-03}$	$7.3 \cdot 10^{-03}$	$8.2 \cdot 10^{-04}$	$4.3 \cdot 10^{-04}$	$1.8 \cdot 10^{-02}$	$5.9 \cdot 10^{-02}$	$1.6 \cdot 10^{-03}$
**4.5**	$7.0 \cdot 10^{-02}$	$5.4 \cdot 10^{-02}$	$2.3 \cdot 10^{-03}$	$6.5 \cdot 10^{-04}$	$6.0 \cdot 10^{-02}$	$2.6 \cdot 10^{-01}$	$3.8 \cdot 10^{-03}$
**4.0**	$1.9 \cdot 10^{-01}$	$2.8 \cdot 10^{-01}$	$7.5 \cdot 10^{-03}$	$1.0 \cdot 10^{-03}$	$3.4 \cdot 10^{-01}$	$7.6 \cdot 10^{-01}$	$1.0 \cdot 10^{-02}$
**3.5**	$4.9 \cdot 10^{-01}$	$1.2 \cdot 10^{00}$	$2.8 \cdot 10^{-02}$	$3.6 \cdot 10^{-03}$	$1.4 \cdot 10^{00}$	$1.7 \cdot 10^{00}$	$5.9 \cdot 10^{-02}$

(PC = Positive control with pH-dependent antigen binding properties, NC = Negative control with non-pH-dependent antigen binding properties, LCS = Light-chain shuffled)

Table S2: Affinity measurements of the Fabs using BLI, related to Figure 2.

Target	Fab ID	pH 7.4			pH 5.5		
		k_{on} ($M^{-1}s^{-1}$)	k_{off} (s^{-1})	K_D (nM)	k_{on} ($M^{-1}s^{-1}$)	k_{off} (s^{-1})	K_D (nM)
**α-cbtx**	TPL0197_01_C08 (Parent)	$1.1 \cdot 10^{4}$	$8.5 \cdot 10^{-4}$	64	$3.8 \cdot 10^{3}$	$5.8 \cdot 10^{-3}$	1500
	TPL0544_01_B01 (LCS)	$6.4 \cdot 10^{3}$	$2.6 \cdot 10^{-4}$	41.1	$2.6 \cdot 10^{3}$	$5.5 \cdot 10^{-4}$	209
	2555_01_A01 (PC)	$1.5 \cdot 10^{4}$	$5.0 \cdot 10^{-4}$	33.8	$4.9 \cdot 10^{4}$	$1.2 \cdot 10^{-2}$	257
	2554_01_D11 (NC)	$1.0 \cdot 10^{4}$	$8.1 \cdot 10^{-4}$	1.3	$4.6 \cdot 10^{4}$	$4.0 \cdot 10^{-4}$	8.7
**M-II**	TPL0039_05_B04 (Parent)	$4.4 \cdot 10^{5}$	$9.2 \cdot 10^{-3}$	20.8	$3.2 \cdot 10^{5}$	$2.4 \cdot 10^{-2}$	76.6
	TPL0552_02_A05 (LCS)	$5.4 \cdot 10^{5}$	$1.3 \cdot 10^{-2}$	24.8	$2.9 \cdot 10^{5}$	$6.7 \cdot 10^{-2}$	226
	TPL0039_05_A03 (NC)	$2.0 \cdot 10^{5}$	$3.1 \cdot 10^{-4}$	1.5	$1.8 \cdot 10^{5}$	$1.1 \cdot 10^{-3}$	6.04

On-rates (k_{on}), off-rates (k_{off}), and affinities (K_D) of the Fabs at pH 7.4 as determined by BLI. (LCS = light-chain shuffled clone, NC = negative control, PC = positive control). Positive control means that the Fab showed a drastically increased off-rate at pH 5.5 compared to pH 7.4. Negative control implies that the off-rates at pH 5.5 and pH 7.4 were similar.

Table S3: Similarity between parental and light-chain shuffled (LCS) antibody variable regions and germline antibody variable regions of the light chain, related to Figure 2.

**Target**	**IgG**	**V region similarity to germline**	**J region similarity to germline**
**α-cbtx**	TPL0197_01_C08 (Parent)	98.96%	77.78%
	TPL0544_01_B01 (LCS)	98.96%	77.78%
**M-II**	TPL0039_05_B04 (Parent)	99.3%	97.37%
	TPL0552_02_A05 (LCS)	97.25%	100%

Table S4: Affinity measurements of the α-cbtx IgGs using SPR, related to Figure 3.

Target	ID	pH 7.4			pH 5.5		
		k_{on} ($M^{-1}s^{-1}$)	k_{off} (s^{-1})	K_D (nM)	k_{on} ($M^{-1}s^{-1}$)	k_{off} (s^{-1})	K_D (nM)
**α-cbtx**	TPL0197_01_C08 (Parent)	$1.32 \cdot 10^5$	$3.05 \cdot 10^{-4}$	2.31	$1.14 \cdot 10^5$	$5.67 \cdot 10^{-3}$	49.8
	TPL0544_01_B01 (LCS)	$1.23 \cdot 10^5$	$2.97 \cdot 10^{-5}$	0.24	$5.60 \cdot 10^4$	$3.10 \cdot 10^{-4}$	5.54
	2555_01_A01 (PC)	$2.22 \cdot 10^6$	$7.98 \cdot 10^{-4}$	0.36	$5.44 \cdot 10^5$	$5.87 \cdot 10^{-3}$	10.8
	2554_01_D11 (NC)	$1.66 \cdot 10^6$	$5.26 \cdot 10^{-5}$	0.032	$6.64 \cdot 10^5$	$1.55 \cdot 10^{-4}$	0.23

On-rates (k_{on}), off-rates (k_{off}), and affinities (K_D) of the IgGs at pH 7.4 as determined by SPR. (LCS = light-chain shuffled clone, NC = negative control, PC = positive control). Positive control means that the IgG showed a drastically increased off-rate at pH 5.5 compared to pH 7.4. Negative control implies that the off-rates at pH 5.5 and pH 7.4 were similar.

Table S5. Amount of IgG1 immobilized onto biosensor chip by amine coupling, related to STAR methods.

**Channel**	**Flow cell**	**Immobilized ligand**	**Amount immobilized (RU)**
1	2	C08	1102.7
2	2	B01	1088.0
3	2	D11	1223.0
4	2	A01	1217.6
